# Household environment associated with anaemia among children aged 6–59 months in Ethiopia: a multilevel analysis of Ethiopia demographic and health survey (2005–2016)

**DOI:** 10.1186/s12889-024-17780-y

**Published:** 2024-01-29

**Authors:** Biniyam Sahiledengle, Lillian Mwanri, Kingsley Emwinyore Agho

**Affiliations:** 1 Department of Public Health, Madda Walabu University Goba Referral Hospital, Bale-Goba, Ethiopia; 2 Research Centre for Public Health Research, Equity and Human Flourishing, Torrens University Australia, Adelaide Campus, Adelaide, SA 5000 Australia; 3 School of Health Sciences, Western Sydney University, Locked Bag 1797, Penrith, NSW 2751 Australia; 4 School of Medicine, Translational Health Research Institute, Western Sydney University, Campbelltown Campus, Penrith, NSW 2571 Australia; 5 African Vision Research Institute, University of KwaZulu-Natal, Durban, 4041 South Africa

**Keywords:** Anaemia, Children, Ethiopia, Open defecation, WASH

## Abstract

**Background:**

Anaemia continues to be a major public health challenge globally, including in Ethiopia. Previous studies have suggested that improved household environmental conditions may reduce anaemia prevalence; however, population-level evidence of this link is lacking in low-income countries. Therefore, this study aimed to examine the association between environmental factors and childhood anaemia in Ethiopia.

**Methods:**

In this study, we conducted an analysis of the data from the Ethiopian Demographic and Health Survey (EDHS), a nationally representative population-based survey conducted in Ethiopia between 2005 and 2016. The study included a total of 21,918 children aged 6–59 months. Children were considered anemic if their hemoglobin (Hb) concentration was less than 11.0 g/dl. To examine the association between environmental factors and anemia, we used multilevel mixed-effect models. These models allowed us to control for various confounding factors including: child, maternal, household and community-level variables. The study findings have been reported as adjusted odds ratios (AORs) along with 95% confidence intervals (CIs) at a significance level of *p* < 0.05.

**Results:**

The study found the overall prevalence of childhood anaemia to be 49.3% (95%CI: 48.7–49.9) between 2005 and 2016 in Ethiopia. The prevalence was 47.6% (95%CI: 46.1–49.1) in 2005, 42.8% (95%CI: 41.8–43.8) in 2011, and increased to 57.4% (95%CI: 56.3–58.4) in 2016. The pooled data showed that children from households practising open defecation were more likely to be anaemic (AOR: 1.19, 95% CI: 1.05–1.36). In our survey specify analysis, the odds of anaemia were higher among children from households practising open defecation (AOR: 1.33, 95% CI: 1.12–1.58) in the EDHS-2011 and EDHS-2016 (AOR: 1.49, 95% CI: 1.13–1.90). In contrast, neither household water sources nor the time to obtain water was associated with anaemia after controlling for potential confounders. The other variables significantly associated with childhood anaemia include: the child’s age (6–35 months), not fully vaccinated (AOR: 1.14, 95%CI: 1.05–1.24), children not dewormed in the last 6 months (AOR: 1.11, 95%CI: 1.01–1.24), children born to mothers not working (AOR: 1.10, 95%CI: 1.02–1.19), children from poor households (AOR: 1.18: 95%CI: 1.06–1.31), and rural residence (AOR: 1.23, 95%CI: 1.06–1.42).

**Conclusion:**

In Ethiopia, about fifty percent of children suffer from childhood anemia, making it a serious public health issue. Open defecation is a major contributing factor to this scourge. To address this issue effectively, it is recommended to strengthen initiatives aimed at eliminating open defecation that involve various approaches, including sanitation infrastructure development, behavior change campaigns, and policy interventions. In addition, to reduce the burden of anemia in children, a multi-faceted approach is necessary, involving both prevention and treatment strategies.

**Supplementary Information:**

The online version contains supplementary material available at 10.1186/s12889-024-17780-y.

## Introduction

Anaemia is a condition marked by low levels of haemoglobin in the blood. However, a broader definition is a lowered ability of the blood to carry oxygen in which the number of red blood cells or the haemoglobin concentration within them is lower than normal [1, 2]. Nutritional iron deficiency has been considered the most common cause of anaemia that particularly affects young children. According to the World Health Organization (WHO) estimate, 42% of children less than 5 years of age worldwide are anaemic, and most of them reside in low-and middle-income countries (LIMCs) [1].

The prevalence of anaemia varies by geographic region [3] across the globe. In Sub-Saharan Africa (SSA), anaemia is one of the leading causes of morbidity and mortality among under-five children [4–7]. A recent estimate from 32 SSA countries reported that the prevalence of anaemia among children aged 6–59 months ranged from 23.7% to 87.9% [6, 8] and 76.6% among children aged 6–23 months [5]. Like many other SSA countries, anaemia is Ethiopia is serious public health problem. According to the Ethiopian Demographic Health Survey (EDHS), the prevalence of anaemia among Ethiopian children was 57% in 2016 [9]. A systematic review and meta-analysis finding also reported a relatively higher prevalence (50.4%) of anaemia among children under 2 years old in Ethiopia [10].

To address undernutrition and micronutrient deficiencies among children, the Government of Ethiopia has developed the National Nutrition Strategy and the National Nutrition Programmes (NNP) [11, 12]. Additional initiatives have embodied the government's commitment to improve nutrition, such as the Seqota Declaration (2015–2030) which aims to eliminate all forms of malnutrition among children by 2030 [13], and the national nutrition strategy target of reducing anaemia to 40% by 2025 [12]. However, the problem of anaemia among children has persisted, and the progress towards reducing this scourge has overall been gradual [9]. For instance, the prevalence of anaemia increased sharply from 44 to 57% among children aged 6–59 months in Ethiopia between 2011 to 2016 [9]. The cause of this recent increments in childhood anaemia is not well-defined [14] and this sharp increase of anaemia may be due to selection bias. Furthermore, the Ethiopian National Nutrition Program's (NPP) second phase (2015–2020) failed to reduce anaemia in children aged 6–59 months from 39 to 24% by 2020, necessitating a recalculation of the national strategy [15].

In its effort to end stunting in children under two years by 2030 [13], the Government of Ethiopia has actively been promoting nutrition specific interventions that address the immediate determinants of malnutrition. Examples of such interventions are the National Nutrition Programmes (NNP) and the National food and nutrition policy (FNP), including the recent “*Seqota Declaration”*[13]. In addition, to improve the nutritional status of children and to achieve the universal access to water, sanitation and hygiene (WASH) services, the government of Ethiopia has introduced several nutrition-sensitive interventions in collaboration with various stakeholders to address the underlying causes of undernutrition and anemia, such as one WASH National Program (OWNP) [16], the Community Led Accelerated WASH (Co-WASH) [17], the Global WASH program [18] and a flagship program to ‘End Open Defecation’[19]. These initiatives and programs play a crucial role in addressing the high burden of childhood undernutrition and the challenges related to WASH in Ethiopia. Over the past decade, Ethiopia has witnessed encouraging progress in the reduction of malnutrition and improvements in access to WASH. However, to achieve a global commitment to tackle undernutrition and to ensure access to water and sanitation for all, specifically the Sustainable Development Goals (SDG 2 and SDG 6), Ethiopia still needs to continue investing significantly in nutrition and WASH [15, 20–22].

Earlier studies in Ethiopia on anaemia were mainly localised [23–27], and the population-based studies on anaemia have focused on the prevalence and socio-demographic determinants [5, 14, 28–30]. Additional studies have mapped anaemia distribution [28, 31, 32] and regional inequalities [33]. However, to our knowledge, no population-level study has examined the association between environmental risk factors (household sanitation facility, type of water source, time spent to get to the water source, biomass fuel used for cooking, and housing status) and childhood anaemia in Ethiopia. Furthermore, current evidence on the association between childhood anaemia and household sanitation or water source has been inconsistent and equivocal [34–40]. Therefore, this analysis aimed to examine the association between environmental factors and childhood anaemia in Ethiopia. Understanding the underlying environmental factors of anaemia is essential for creating evidence that can inform the development of successful interventions that target context-specific anaemia control programs and strategies that could help improve the current programs.

## Methods

### Data source and study design

Data from the Ethiopian Demographic and Health Survey (EDHS) from 2005, 2011 and 2016 rounds were analysed. The EDHS is a cross-sectional, nationally representative survey. EDHS employs a two-stage multistage, sampling weights and cluster sampling method to establish a representative sample of households at the national and regional levels [21, 41, 42]. The complete sampling procedure has been explained in the final reports of 2005, 2011, and 2016 EDHS [21, 41, 42]. The EDHS first recorded childhood anaemia in the second survey; our analysis was restricted to EDHS data (2005–2016). The EDHS collected blood samples among all children aged 6 to 59 months included in the survey for haemoglobin tests using a battery-operated portable HemoCue analyser (*HemoCue®*). A total of 21,918 (weighted data) children with complete haemoglobin records were included in this study (EDHS-2005, *n* = 4,259; EDHS-2011, *n* = 9,259; EDHS-2016, *n* = 8,399).

### Outcome variable

The study’s dependent variable was childhood anaemia. Anaemia was described based on the WHO cutoff point, and a haemoglobin level of less than 11 g/deciliter (g/dl) for children aged 6–59 months old was categorised as anaemic coded as ‘1’ otherwise as ‘0’.

### Independent variables

The main exposure variable was environmental factors (with respective categories) include household sanitation facility (improved, unimproved, and open defecation), source of drinking water (improved, unimproved), type of cooking fuel (clean fuels, solid fuels), housing status (built from finished materials, built from natural/unfinished materials), and time to get a water source (on-premises, ≤ 30 min round-trip fetching times, 31–60 min round-trip fetching times, and over 60 min round-trip fetching times) (Table 1).

**Table 1:** Description and classification of environmental factors used in the study

Variables	Variable type and category	Measurement/Definition
Toilet facility^a^	Categorical data is categorised as “Improved", "Unimproved", or "Open defecation."	Based on the WHO definition, facilities would be considered improved if any of the following occurred: flush/pour flush toilets to piped sewer systems, septic tanks, and pit latrines; ventilated improved pit (VIP) latrines; pit latrines with slabs; and composting toilets. Unimproved sanitation included: flush or pour-flush to elsewhere; pit latrine without a slab or open pit; bucket, hanging toilet, or hanging latrine. Other facilities, including households with no facility or use of bush/field, were considered as open defecation
Source of drinking water^a^	Categorical data, categorised as "Improved" or "Unimproved."	Improved sources of drinking water included piped water, public taps, standpipes, tube wells, boreholes, protected dug wells and springs, and rainwater. Other sources of drinking water were regarded as unimproved
Time to get a water source	Categorical data, categorised as "On-premises", " ≤ 30 min round-trip fetching times", "31–60 min round-trip fetching times", and “over 60 min round-trip fetching times”	Time to obtain drinking water (round trip) was categorised as water on premises, less than 30 min, 30–60 min, or over 60 min
Biomass fuel used for cooking	Categorical data were categorised as "clean fuels" and "solid fuels."	Clean fuels included electricity, liquefied petroleum gas (LPG), and natural gas, while charcoal, firewood, grass/straw, dung, shrubs, and agricultural crop waste represented unclean/solid fuels
Housing status	Categorical data categorise as "built from finished materials" and "built from natural or unfinished materials."	We classified houses as ‘built from finished materials’ if all of the three variables of the wall, roof, and floor materials were finished and otherwise as ‘built from natural or unfinished materials’

^a^The source of drinking water and toilet facilities under the WHO/UNICEF Joint Monitoring Programme

The control variables used in this study were generated based on literature [34, 37] and their availability in the EDHS dataset. The identified factors were categorised into child, maternal, household, and community-level factors. Child factors consisted of the sex of the child (male, female), age of the child (6–11 months, 12–23, 24–35, and 36–59 months), the perceived size of the child at birth (large, average and small), currently breastfeeding (yes, no), had diarrhoea in the last 2 weeks (yes, no), full vaccination (yes, no), received deworming medication in the last 6 month (yes, no), received iron supplementation in the last 6 months (yes, no), received vitamin A last 6 months (yes, no). Maternal factors include mother's age (< 18, 18–24, 25–34, or >  = 35), mother's education (no education, primary and above), mother's occupation (not working, non-agriculture, or agriculture), maternal stature (≥ 155 cm), short (145 to 154.9 cm), very short (< 145 cm), and mother’s body mass index (BMI) (kg/m^2^) (< 18.5 kg/m^2^, 18.5 to 24.9 kg/m^2^, and 25 + kg/m^2^). The household factor included in our analysis was the wealth index (poor, middle, and rich). Community-level factors include the place of residence (rural, urban) and contextual regions (agrarian, pastoralist, or metropolises/city administrations).

### Data analysis

All analyses used STATA/MP version 14.1 (Stata Corp, College Station, TX, USA). The *'Svy'* commands were employed to allow for adjustments for the cluster-sampling design and weight. We conducted frequency tabulations to describe the data used in the study and the distributions of anaemia by background characteristics. The EDHS data are hierarchical, i.e. children are nested within households, and households are nested within clusters (or enumeration areas (EAs)). The use of flat models could underestimate standard errors of the effect sizes, which consequently could affect the decision on null hypothesis. With such data, children within a cluster may have been more similar to each other than children in the rest of the clusters. This violates the assumption of independence of observations and equal variance across the clusters. Hence, we estimated a two-level model with the child data as level 1 and clusters as level 2 [43]. Following that, a hierarchical multilevel model was run in accordance with suggestions made by an earlier study, which suggests a complex hierarchical relationship of many determinants at different levels. This method has made it possible to adequately explore distal factors without being hampered by proximal ones. A similar approach has been used to identify previous related literature [44–46].

First, a bivariable multilevel analysis was performed to identify factors and variables associated with childhood anaemia. Then, we performed a five-stage model as part of the hierarchical multivariable multilevel logistic regression analysis to explore the relationship between household environmental factors and anaemia among children 6–59 months in Ethiopia. The empty model (or null model) was fitted without explanatory variables to estimate random variation in the intercept and the intra-cluster correlation coefficient (ICC; i.e., to evaluate the extent of the cluster variation in childhood anemia). The first model (Model 1) includes the exposure variables-environmental factors were included to identify their association with anaemia. Model 2 contained child-related factors. Model 3 controlled for maternal-related factors. Model 4 and Model 5 controlled for the household and community-level factors, respectively. Variables with a *p*-value less than 0.25 in each stage model were entered in consecutive models. Multicollinearity among the independent variables was checked before their inclusion in the final regression model. Multicollinearity among independent variables was assessed by using the variance inflation factor (VIF). The VIF of 5 has been recommended as the maximum level [47–49]. The intraclass correlation coefficient (ICC) was estimated to determine cluster variability when using a multilevel approach for analyses [50]. The findings of the study were reported as adjusted odds ratios (AORs) along with 95% confidence intervals (CIs) at a significance level of *p* < 0.05. The AORs represent the strength and direction of the association between each environmental factor and the likelihood of anemia in children.

## Results

### Characteristics of the study population

Table 2 illustrates the socio‐demographic characteristics of the sample population and the prevalence of anaemia by characteristics of the study population from 2005 to 2016. In this analysis, a weighted data of 21,918 under-five children participated in the study: 51.2% were male, and 45.9% were 36–59 months. The median age of the study participants was 30 months (Interquartile range (IQR): 14–45 months). A total of 45.6% of mother–child were from the relatively poor wealth category. Almost 46% of children were from households that practised open defecation, 53.8% used unimproved sources of drinking water, and the majority (98.6%) used solid cooking fuel. Throughout the survey years, the prevalence of anaemia was higher in children from households that practised open defecation in 2005 (48.4%), 2011 (53.8%), and 2016 (66.7%).

**Table 2:** Socio‐demographic characteristics of the sample population and prevalence of anaemia among children 6–59 months by characteristics of the study population, EDHS 2005–2016 (*n* = 21,918)

Variables	Weighted, (n)	Weighted, (%)	Children with anaemia, n (%)	Prevalence of anaemia, 95%CI			
				**EDHS-2005**	**EDHS-2011**	**EDHS-2016**	**Pooled (2005–2016)**
***Environmental factors***							
**Sanitation facility**							
Improved	2,140	10.1	948 (8.9)	36.0 (31.4–40.9)	38.6 (36.1–41.1)	58.5 (55.7–61.2)	46.2 (44.4–47.9)
Unimproved	9,591	44.3	4,572 (42.8)	44.5 (41.3–47.7)	40.0 (38.3–41.8)	53.7 (51.9–55.4)	46.7 (45.5–47.9)
Open defecation	9,878	45.6	5,162 (48.3)	48.4 (46.5–50.4)	53.8 (52.4–55.3)	66.7 (65.1–68.3)	56.7 (55.7–57.6)
**Type of cooking fuel**							
Clean fuels	298	1.4	135 (1.3)	46.3 (10.4–86.5)	24.7 (13.9–40.1)	48.9 (42.8–55.2)	41.8 (37.2–46.5)
Solid fuels	21,359	98.6	10,547 (98.7)	47.6 (46.1–49.1)	42.7 (41.7–43.8)	57.7 (56.6–58.8)	51.9 (51.3–52.7)
**Housing status**							
Built from finished materials	452	2.1	175 (1.6)	26.8 (18.9–36.5)	34.1 (29.7–38.7)	56.1 (51.5–60.4)	43.8 (40.7–46.8)
Built from natural or unfinished materials	21,458	97.9	10,638 (98.4)	46.7 (45.1–48.3)	47.5 (46.5–48.6)	60.4 (59.3–61.5)	52.1 (51.4–52.8)
**Source of drinking water**							
Improved	10,006	46.2	4,788 (44.8)	46.5 (44.7–48.2)	42.0 (40.3–43.8)	57.7 (55.9–59.5)	48.6 (47.5–49.6)
Unimproved	11,643	53.8	5,893 (55.2)	45.5 (42.0–49.0)	49.5 (48.2–50.8)	61.6 (60.2–62.9)	54.3 (53.4–55.2)
**Time to get a water source**							
On-premises	1,537	7.0	637 (5.9)	31.7 (26.3–37.6)	38.7 (35.4–42.1)	54.6 (51.7–57.4)	46.2 (44.1–48.3)
≤ 30 min	12,282	56.0	6,046 (55.9)	46.3 (44.2–48.4)	43.7 (42.2–45.2)	59.4 (57.7–60.9)	49.9 (48.9–50.9)
31–60 min	4,663	21.3	2,385 (22.1)	47.6 (43.8–51.4)	47.2 (44.9–49.5)	62.7 (60.1–65.1)	53.1 (51.5–54.6)
> 60 min	3,435	15.7	1,745 (16.1)	50.1 (46.4–53.7)	56.4 (54.3–58.6)	64.2 (61.7–66.8)	57.9 (56.4–59.4)
***Child factors***							
**Sex**							
Male	11,220	51.2	5,547 (51.3)	47.4 (45.2–49.6)	47.3 (45.8–48.7)	60.6 (59.0–62.1)	52.3 (51.4–53.3)
Female	10,698	48.8	5,267 (48.7)	45.1 (42.8–47.3)	46.5 (45.0–47.9)	59.7 (58.1–61.2)	51.1 (50.1–52.1)
**Age (in categories)**							
6–11	2,481	11.3	1,676 (15.5)	56.2 (51.5–60.8)	61.9 (58.9–64.9)	77.9 (75.0–80.6)	66.7 (64.7–68.6)
12–23	4,716	21.6	2,961 (27.4)	60.1 (56.8–63.3)	59.5 (57.2–61.7)	72.6 (70.4–74.6)	64.8 (63.4–66.2)
24–35	4,629	21.2	2,308 (21.4)	44.5 (41.2–47.9)	49.6 (47.4–51.8)	62.7 (60.4–65.0)	53.6 (52.1–55.1)
36–59	10,028	45.9	3,851 (35.7)	37.8 (35.6–40.1)	36.4 (34.9–37.9)	48.4 (46.7–50.1)	41.0 (40.0–42.0)
**Birth interval**							
< 33 months	15,046	68.6	7,594 (70.2)	45.9 (44.0–47.8)	47.5 (46.3–48.7)	62.2 (60.9–63.5)	52.7 (51.9–53.5)
≥ 33 months	6,872	31.4	3,219 (29.8)	46.9 (44.2–49.7)	45.5 (43.6–47.4)	55.3 (53.2–57.3)	49.4 (48.1–50.6)
**Size of a child at birth**							
Larger	7,029	32.2	3,532 (32.7)	47.8 (45.0–50.7)	45.7 (43.8–47.6)	57.8 (55.8–59.8)	50.7 (49.4–51.9)
Average	8,867	40.6	4,229 (39.2)	45.8 (43.4–48.3)	44.9 (43.3–46.5)	58.6 (56.9–60.3)	50.4 (49.3–51.5)
Small	5,950	27.2	3,025 (28.1)	45.2 (42.2–48.2)	50.7 (48.9–52.6)	65.6 (63.5–67.7)	54.9 (53.6–56.1)
**Currently breastfeeding**							
Yes	15,491	70.7	8,102 (74.9)	48.9 (47.0–50.7)	49.8 (48.5–51.1)	62.5 (61.2–63.9)	54.2 (53.4–55.0)
No	6,427	29.3	2,712 (25.1)	39.5 (36.7–42.4)	41.1 (39.3–42.8)	55.8 (54.0–57.7)	46.8 (45.6–47.9)
**Full vaccination**							
Yes	4,255	24.5	2,013 (22.9)	43.4 (39.5–47.2)	40.0 (38.0–41.9)	61.9 (59.5–64.3)	48.1 (46.6–49.5)
No	13,129	75.5	6,781 (77.1)	47.7 (45.9–49.5)	49.8 (48.5–51.0)	73.7 (72.0–75.4)	54.6 (53.7–55.5)
**Diarrhoea**							
Yes	3,199	14.6	1,776 (16.4)	54.2 (50.5–57.8)	54.7 (52.1–57.2)	63.9 (60.8–66.9)	57.4 (55.6–59.1)
No	18,688	85.4	9,028 (83.6)	44.5 (42.8–46.2)	45.4 (44.3–46.6)	59.5 (58.4–60.7)	50.7 (50.0–51.5)
**Received deworming medication in the last 6 months**							
Yes	3,037	13.8	1,303 (12.1)	-	39.9 (37.7–42.2)	53.6 (50.6–56.7)	44.8 (43.0–46.7)
No	18,881	86.1	9,510 (87.9)	46.2 (44.7–47.8)	48.6 (47.5–49.8)	61.1 (59.9–62.3)	52.8 (52.1–53.5)
**Iron supplementation**							
Yes	1,356	6.2	652 (6.0)	-	46.6 (43.2–50.1)	55.4 (51.4–59.3)	50.4 (47.8–52.9)
No	20,562	93.8	10,161 (94.0)	46.2 (44.7–47.8)	46.9 (45.8–48.0)	60.5 (59.4–61.7)	51.8 (51.1–52.5)
**Vitamin A last 6 months**							
Yes	10,834	50.4	5,106 (48.1)	46.8 (44.5–49.2)	43.6 (42.3–45.0)	56.9 (55.3–58.6)	48.9 (48.0–49.9)
No	10,681	49.6	5,509 (51.9)	45.4 (43.3–47.6)	50.9 (49.4–52.5)	63.1 (61.6–64.7)	54.6 (53.6–55.5)
***Parental factors***							
**Mother's age**							
< 18	106	0.5	56 (0.5)	52.9 (29.6–75.1)	49.0 (35.6–62.6)	77.8 (61.2–88.6)	59.6 (49.9–68.6)
18–24	4,668	21.3	2,452 (21.3)	47.6 (44.3–50.9)	48.9 (46.7–51.1)	64.3 (62.0–66.4)	54.4 (53.0–55.9)
25–34	11,536	52.6	5,684 (52.6)	45.3 (43.1–47.5)	46.8 (45.4–48.2)	60.5 (59.0–62.0)	51.7 (50.7–52.7)
≥ 35	5,609	25.6	2,621 (25.6)	46.7 (43.7–49.8)	45.3 (43.2–47.3)	55.1 (52.8–57.3)	49.1 (47.8–50.5)
**Mother's education**							
No education	15,457	70.5	7,730 (71.5)	47.2 (45.4–49.0)	49.4 (48.1–50.4)	62.5 (61.1–63.8)	53.5 (52.7–54.3)
Primary and above	6,461	29.5	3,083 (28.5)	42.9 (39.7–46.2)	41.0 (39.2–42.9)	55.7 (53.8–57.6)	47.6 (46.4–48.9)
**Mother's currently working**							
Yes	6,674	30.5	3,137 (29.0)	48.0 (44.8–51.2)	41.5 (39.7–43.4)	53.9 (51.8–55.9)	47.1 (45.8–48.4)
No	15,238	69.5	7,675 (71.0)	45.6 (43.8–47.5)	49.2 (48.0–50.5)	62.6 (61.3–63.8)	53.5 (52.7–54.4)
**Maternal stature**							
Normal/Tall (> = 155 cm)	13,517	61.7	6,663 (61.6)	46.0 (44.1–47.9)	46.6 (45.3–47.9)	61.2 (59.8–62.5)	52.1 (51.3–53.0)
Short (145 to 154.9 cm)	7,860	35.9	3,861 (35.7)	47.3 (44.6–50.1)	47.6 (45.8–49.3)	57.1 (55.1–59.1)	50.9 (49.7–52.1)
Very short (< 145 cm)	540	2.5	289 (2.7)	36.7 (27.3–47.4)	44.4 (37.6–51.4)	71.3 (63.1–78.3)	51.5 (46.7–56.3)
**Maternal BMI (kg/m**^**2**^**)**							
< 18.5	4,608	21.0	2,437 (22.5)	50.8 (47.6–54.1)	52.0 (50.1–54.0)	65.4 (63.2–67.5)	56.7 (55.3–58.0)
18.5 to 24.9	16,263	74.2	7,909 (73.2)	45.3 (43.4–47.1)	45.7 (44.4–46.9)	58.6 (57.3–59.9)	50.4 (49.5–51.2)
25 +	1,034	4.7	463 (4.3)	37.5 (30.5–45.2)	37.1 (33.1–41.4)	56.5 (52.6–60.2)	46.6 (43.9–49.3)
**Listening to radio**							
Yes	8,246	37.6	3,824 (35.4)	42.8 (40.2–45.5)	44.6 (43.0–46.2)	54.2 (51.9–56.5)	46.7 (45.6–47.9)
Not at all	13,665	62.4	6,985 (64.4)	48.0 (46.1–49.9)	48.6 (47.3–50.0)	61.9 (60.7–63.2)	54.3 (53.5–55.1)
**Watching television**							
Yes	4,738	21.6	2,076 (19.2)	32.3 (27.9–37.1)	39.0 (37.2–40.9)	53.3 (50.7–55.8)	43.1 (41.6–44.5)
Not at all	17,163	78.4	8,729 (80.8)	47.9 (46.2–49.6)	50.2 (48.9–51.4)	61.8 (60.5–62.9)	54.2 (53.4–54.9)
***Household factors***							
**Wealth index**							
Poor	10,002	45.6	5,352 (49.5)	49.5 (47.2–51.8)	52.3 (50.8–53.7)	66.5 (65.1–67.9)	57.5 (56.6–58.5)
Middle	4,624	21.1	2,224 (20.6)	49.3 (45.5–53.0)	44.2 (41.6–46.8)	53.1 (50.2–55.9)	48.4 (46.7–50.1)
Rich	7,292	33.3	3,237 (29.9)	40.6 (38.0–43.2)	40.5 (38.8–42.2)	52.3 (50.3–54.3)	44.5 (43.4–45.7)
***Community-level characteristics***							
**Residence**							
Urban	2,326	10.6	915 (8.5)	32.5 (28.6–36.6)	37.1 (34.7–39.6)	54.2 (51.5–56.9)	43.2 (41.5–44.8)
Rural	19,592	89.4	9,898 (91.5)	48.4 (46.7–50.0)	48.8 (47.7–49.9)	61.3 (60.1–62.5)	53.4 (52.6–54.1)
**Region**							
Agrarian	11,947	54.5	5,209 (48.1)	46.4 (44.3–48.4)	41.9 (40.7–43.3)	56.1 (54.7–57.6)	48.1 (47.3–49.1)
Pastoralist	9,456	43.1	5,392 (49.9)	48.4 (45.4–51.5)	59.3 (57.2–61.3)	68.3 (66.2–70.3)	60.5 (59.2–61.9)
City administration	515	2.3	213 (1.9)	41.3 (37.1–45.6)	46.2 (43.6–48.8)	63.1 (60.1–66.0)	51.3 (49.5–53.0)

### Prevalence of anaemia

**Fig. 1:**
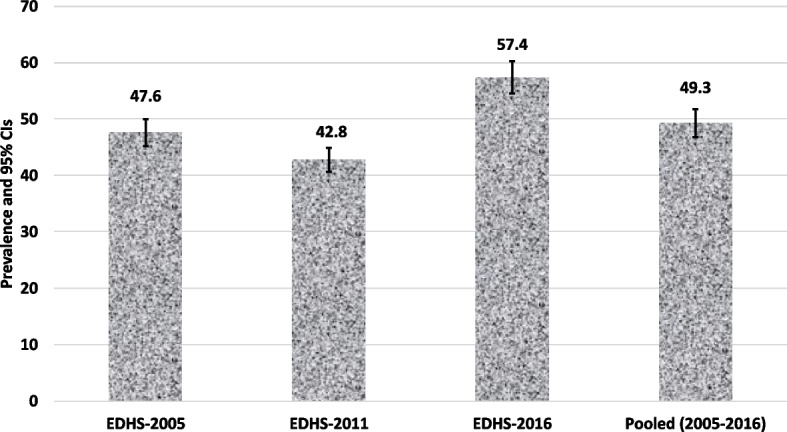
Prevalence of anaemia in children aged 6–59 months in Ethiopia (2005–2016)

As shown in Fig. 1, the prevalence of anaemia in children aged 6–59 months was 47.6% (95%CI: 46.1–49.1) in 2005, 42.8% (95%CI: 41.8–43.8) in 2011 and increased to 57.4% (95%CI: 56.3–58.4) in 2016. Overall, 49.3% (95%CI: 48.7–49.9) of children were anaemic.

### Factors associated with anaemia

Tables 3 and 4 present results for the bivariable and multivariable multilevel analysis of background characteristics with anaemic children 6–59 months in Ethiopia, respectively. After adjusting for potential confounders, household sanitation facilities remained an independent predictor of anaemia, and children from households practising open defecation were more likely to be anaemic than their counterparts (AOR: 1.19, 95% CI: 1.05–1.36) (Table 4).

**Table 3:** Unadjusted association between anaemia and environmental factors and other study covariates among children aged 6–59 months in Ethiopia, EDHS 2005–2016 (*n* = 21,918)

Variables	Children with anaemia (%)	Unadjusted OR (95%CI)	*p*-value
***Environmental factors***			
**Sanitation facility**			
Improved	948 (8.9)	Ref	
Unimproved	4,572 (42.8)	1.09 (0.99–1.19)	0.059
Open defecation	5,162 (48.3)	1.52 (1.39–1.67)	*p* < 0.001
**Type of cooking fuel**			
Clean fuels	135 (1.3)	Ref	
Solid fuels	10,547 (98.7)	1.34 (1.09–1.64)	0.005
**Housing status**			
Built from finished materials	175 (1.6)	Ref	
Built from natural or unfinished materials	10,638 (98.4)	1.38 (1.20–1.58)	*p* < 0.001
**Source of drinking water**			
Improved	4,788 (44.8)	Ref	
Unimproved	5,893 (55.2)	1.24 (1.16–1.32)	*p* < 0.001
**Time to get a water source**			
On-premise	637 (5.9)	Ref	
≤ 30 min	6,046 (55.9)	1.13 (1.02–1.25)	0.017
31–60 min	2,385 (22.1)	1.27 (1.14–1.43)	*p* < 0.001
> 60 min	1,745 (16.1)	1.48 (1.32–1.66)	*p* < 0.001
***Child factors***			
**Sex**			
Male	5,547 (51.3)	Ref	
Female	5,267 (48.7)	0.95 (0.90–1.01)	0.092
**Age (months)**			
6–11	1,676 (15.5)	3.09 (2.79–3.41)	*p* < 0.001
12–23	2,961 (27.4)	2.81 (2.60–3.04)	*p* < 0.001
24–35	2,308 (21.4)	1.72 (1.59–1.85)	*p* < 0.001
36–59	3,851 (35.7)	Ref	
**Birth interval**			
7- 33 months	7,594 (70.2)	Ref	
≥ 33 months	3,219 (29.8)	0.88 (0.83–0.94)	*p* < 0.001
**Size of a child at birth**			
Larger	3,532 (32.7)	Ref	
Average	4,229 (39.2)	0.99 (0.93–1.07)	0.995
Small	3,025 (28.1)	1.17 (1.08–1.26)	*p* < 0.001
**Currently breastfeeding**			
Yes	8,102 (74.9)	Ref	
No	2,712 (25.1)	0.71 (0.67–0.76)	*p* < 0.001
**Full vaccination**			
Yes	2,013 (22.9)	Ref	
No	6,781 (77.1)	1.25 (1.16–1.34)	*p* < 0.001
**Diarrhoea**			
Yes	1,776 (16.4)	1.36 (1.25–1.47)	*p* < 0.001
No	9,028 (83.6)	Ref	
**Received deworming medication in the last 6 months**			
Yes	1,303 (12.1)	Ref	
No	9,510 (87.9)	1.36 (1.25–1.49)	*p* < 0.001
**Iron supplementation**			
Yes	652 (6.0)	Ref	
No	10,161 (94.0)	1.07 (0.95–1.19)	0.248
**Vitamin A last 6 months**			
Yes	5,106 (48.1)	Ref	
No	5,509 (51.9)	1.22 (1.15–1.30)	*p* < 0.001
***Parental factors***			
**Mother's age**			
15–18	56 (0.5)	1.41 (0.94–2.13)	0.096
18–24	2,452 (21.3)	1.23 (1.13–1.34)	*p* < 0.001
25–34	5,684 (52.6)	1.09 (1.02–1.78)	0.008
35–49	2,621 (25.6)	Ref	
**Mother's education**			
No education	7,730 (71.5)	1.21 (1.14–1.29)	*p* < 0.001
Primary and above	3,083 (28.5)	Ref	
**Mother's currently working**			
Yes	3,137 (29.0)	Ref	
No	7,675 (71.0)	1.27 (1.19–1.35)	*p* < 0.001
**Maternal stature**			
Normal/Tall (> = 155 cm)	6,663 (61.6)	Ref	
Short (145 to 154.9 cm)	3,861 (35.7)	0.98 (0.92–1.04)	0.500
Very short (< 145 cm)	289 (2.7)	1.01 (0.82–1.24)	0.901
**Maternal BMI (kg/m**^**2**^**)**			
< 18.5	2,437 (22.5)	Ref	
18.5 to 24.9	7,909 (73.2)	0.79 (0.74–0.84)	*p* < 0.001
25 +	463 (4.3)	0.68 (0.60–0.78)	*p* < 0.001
**Listening to radio**			
Yes	3,824 (35.4)	Ref	
Not at all	6,985 (64.4)	1.35 (1.27–1.44)	*p* < 0.001
**Watching television**			
Yes	2,076 (19.2)	Ref	
Not at all	8,729 (80.8)	1.51 (1.41–1.63)	*p* < 0.001
***Household factors***			
**Wealth index**			
Poor	5,352 (49.5)	1.68 (1.56–1.79)	*p* < 0.001
Middle	2,224 (20.6)	1.21 (1.10–1.32)	*p* < 0.001
Rich	3,237 (29.9)	Ref	
***Community-level characteristics***			
**Residence**			
Urban	915 (8.5)	Ref	
Rural	9,898 (91.5)	1.56 (1.43–1.70)	*p* < 0.001
**Region**			
Agrarian	5,209 (48.1)	Ref	
Pastoralist	5,392 (49.9)	1.61 (1.49–1.74)	*p* < 0.001
City administration	213 (1.9)	1.05 (0.96–1.16)	0.281
**EDHS**			
2005	2,029 (18.8)	0.56 (0.51–0.61)	*p* < 0.001
2011	3,964 (36.7)	0.55 (0.52–0.59)	*p* < 0.001
2016	4,820 (44.5)	Ref

**Table 4:** Adjusted association between anaemia and environmental factors and other study covariates among children 6–59 months in Ethiopia, EDHS 2005–2016 (*n* = 21,918)

Variables	Model 0	Model 1	Model 2	Model 3	Model 4	Model 5
	**Null model**	**AOR(95%CI)**	**AOR (95%CI)**	**AOR (95%CI)**	**AOR (95%CI)**	**AOR (95%CI)**
***Environmental factors***						
**Sanitation facility**						
Improved		Ref	Ref	Ref	Ref	Ref
Unimproved		1.05 (0.94–1.16)	1.03 (0.91–1.16)	0.98 (0.87–1.11)	0.98 (0.87–1.11)	0.93 (0.82–1.05)
Open defecation		1.41 (1.27–1.56)**	1.43 (1.27–1.60)**	1.29 (1.15–1.46)	1.21 (1.06–1.36)*	1.19 (1.05–1.36)*
**Source of drinking water**						
Improved		Ref	Ref	Ref	Ref	Ref
Unimproved		1.17 (1.09–1.24)**	1.16 (1.08–1.25)**	1.13 (1.05–1.22)*	1.09 (1.01–1.18)*	1.03 (0.95–1.12)
**Time to get a water source**						
On-premise		Ref	Ref	Ref	Ref	Ref
≤ 30 min		0.91 (0.81–1.02)	0.94 (0.82–1.08)	0.86 (0.75–0.98)*	0.85 (0.74–0.98)*	0.98 (0.85–1.13)
31–60 min		0.99 (0.87–1.13)	1.04 (0.89–1.21)	0.95 (0.81–1.11)	0.94 (0.81–1.09)	1.04 (0.88–1.23)
> 60 min		1.14 (0.99–1.30)	1.23 (1.05–1.44)*	1.09 (0.93–1.29)	1.08 (0.93–1.26)	1.17 (0.98–1.38)
**Housing status**						
Built from finished materials		Ref	Ref	Ref		
Built from natural or unfinished materials		1.11 (0.95–1.29)	1.18 (0.98–1.42)	1.10 (0.91–1.33)		
**Type of cooking fuel**						
Clean fuels		Ref				
Solid fuels		1.06 (0.85–1.32)				
***Child factors***						
**Sex**						
Male			Ref	Ref	Ref	Ref
Female			0.95 (0.89–1.02)	0.95 (0.89–1.02)	0.96 (0.89–1.02)	0.96 (0.89–1.02)
**Age (months)**						
6–11			3.62 (3.23–4.06)**	3.63 (3.25–4.07)	3.61 (3.22–4.03)**	2.94 (2.62–3.30)**
12–23			3.38 (3.08–3.70)**	3.38 (3.09–3.69)	3.39 (3.10–3.71)**	2.70 (2.46–2.97)**
24–35			2.07 (1.90–2.26)**	2.06 (1.89–2.25)	2.06 (1.89–2.24)**	1.62 (1.48–1.77)**
36–59			Ref	Ref	Ref	Ref
**Size of a child at birth**						
Larger			Ref	Ref		
Average			0.99 (0.91–1.07)	0.97 (0.89–1.05)		
Small			1.06 (0.97–1.15)	1.03 (0.94–1.13)		
**Full vaccination**						
Yes			Ref	Ref	Ref	Ref
No			1.12 (1.03–1.22)*	1.09 (1.01–1.18)*	1.08 (1.00–1.18)*	1.14 (1.05–1.24)*
**Received deworming medication in the last 6 months**						
Yes			Ref	Ref	Ref	Ref
No			1.12 (1.01–1.25)*	1.10 (0.99–1.22)	1.11 (0.99–1.23)	1.11 (1.01–1.24)*
**Iron supplementation**						
Yes			0.88 (0.77–1.02)	0.90 (0.78–1.04)	0.91 (0.78–1.04)	0.95 (0.82–1.09)
No			Ref	Ref	Ref	Ref
**Vitamin A last 6 months**						
Yes			Ref	Ref	Ref	Ref
No			1.09 (1.02–1.18)*	1.06 (0.99–1.14)	1.06 (0.98–1.14)	1.02 (0.94–1.09)
**Currently breastfeeding**						
Yes			Ref			
No			1.01 (0.93–1.08)			
**Birth interval**						
7- 33 months			Ref			
≥ 33 months			0.99 (0.92–1.06)			
**Diarrhoea**						
Yes			1.05 (0.95–1.15)			
No			Ref			
***Parental factors***						
**Mother's age**						
15–18				0.73 (0.47–1.14)	0.73 (0.47–1.14)	0.71 (0.45–1.11)
18–24				1.04 (0.94–1.15)	1.04 (0.94–1.14)	1.01 (0.91–1.11)
25–34				1.05 (0.97–1.14)	1.05 (0.97–1.15)	1.03 (0.94–1.12)
35–49				Ref	Ref	Ref
**Mother's education**						
No education				1.04 (0.96–1.14)		
Primary and above				Ref		
**Mother's currently working**						
Yes				Ref	Ref	Ref
No				1.11 (1.03–1.20)*	1.11 (1.03–1.19)*	1.10 (1.02–1.19)*
**Maternal BMI (kg/m**^**2**^**)**						
< 18.5				Ref	Ref	Ref
18.5 to 24.9				0.84 (0.77–0.90)**	0.85 (0.78–0.91)	0.84 (0.78–0.91)**
25 +				0.84 (0.73–1.03)	0.86 (0.73–1.01)	0.82 (0.69–0.96)*
**Listening to radio**						
Yes				Ref	Ref	Ref
Not at all				1.12 (1.04–1.21)*	1.08 (1.01–1.18)*	1.04 (0.95–1.12)
**Watching television**						
Yes				Ref	Ref	Ref
Not at all				1.27 (1.15–1.41)**	1.26 (1.14–1.39)**	1.27 (1.14–1.40)**
***Household factors***						
**Wealth index**						
Poor					1.27 (1.15–1.40)**	1.18 (1.06–1.31)*
Middle					1.05 (0.94–1.17)	1.01 (0.90–1.13)
Rich					Ref	Ref
***Community-level characteristics***						
**Residence**						
Urban						Ref
Rural						1.23 (1.06–1.42)*
**Region**						
Agrarian						Ref
Pastoralist						1.58 (1.44–1.74)**
City administration/ Metropolis/						1.43 (1.27–1.62)**
**EDHS**						
2005						0.45 (0.40–0.51)**
2011						0.50 (0.46–0.55)**
2016						Ref
**Random effect**						
ICC (%)	6.38	5.48	5.02	4.86	4.93	4.24
Log-likelihood	-14060.391	-13799.074	-10389.692	-10325.692	-10360.817	-10177.156

AOR (Adjusted Odds Ratio), *LL* Log-likelihood

Model 0: Empty model with no independent variables

Model 1: All environmental factors were included in the model

Model 2: Environmental factors (from model 1 with *p* < 0.25) + Child-related factors (from model 0 with *p* < 0.25)

Model 3: Environmental factors (from model 2 with *p* < 0.25) + Child-related factors (from model 2 with *p *< 0.25) + Maternal factors (from model 0 with *p *< 0.25)

Model 4: Environmental factors (from model 3 with *p* < 0.25) + Child-related factors (from model 3 with *p* < 0.25) + Maternal factors (from model 3 with *p* < 0.25) + Household factors (from model 0 with p < 0.25)

Model 5: Environmental factors (from model 4 with *p* < 0.25) + Child related factors (from model 4 with *p* < 0.25) + Maternal factors (from model 4 with *p* < 0.25) + Household factors (from model 4 with *p* < 0.25) + Community level factors (from model 0 with *p* < 0.25)

^*^p-value < 0.05

^**^p < 0.001

Control variables associated with anaemia include: children aged 6–11 months (AOR: 2.94, 95%: 2.62–3.30), 12–24 months (AOR: 2.70, 95% CI: 2.46–2.97), and 24–35 months (AOR: 1.62, 95% CI: 1.48–1.77) were more likely to experience anaemia compared to those aged 36–59 months. The odds of anaemia were higher among children who had not been fully vaccinated (AOR: 1.14, 95%CI: 1.05–1.24) and those who had not received deworming medication in the last 6 month (AOR: 1.11, 95%CI: 1.01–1.24). Anaemia was higher among children born to mothers not working (AOR: 1.10, 95%CI: 1.02–1.19) than their counterparts. On the other hand, the odds of anaemia were lower among children born to mothers with normal BMI (AOR: 0.84, 95%CI: 0.78–0.91) and those overweight/obese (AOR: 0.82, 95%CI: 0.69–0.96) than children born to underweight mothers. Anaemia was more likely to occur among households with a relatively poor wealth index category (AOR: 1.18: 95%CI: 1.06–1.31) than among the rich. The odds of anaemia were higher among rural residents compared to urban (AOR: 1.23, 95%CI: 1.06–1.42). Children who lived in pastoralist areas (AOR: 1.58, 95%CI: 1.44–1.74) and in metropolis (AOR: 1.43, 95%CI 1.27–1.62) were more likely to suffer from anaemia compared to those who lived in the agrarian region. Children included from EDHS-2005 (AOR: 0.45, 95%CI: 0.40–0.51) and EDHS-2011 (AOR: 0.50, 95%CI: 0.46–0.55) were lower odds of having anaemia than those from EDHS-2016 (Table 4 and Supplementary File 1).

Supplementary Files 2, 3, and 4 present 2005, 2011, and 2016 surveys specific to the multilevel multivariable analysis of anaemia and environmental factors in detail. In the 2011 EDHS, anaemia was more likely to occur among children from households practising open defecation (AOR: 1.33, 95% CI: 1.12–1.58) (Table 5 and Supplementary Files 3). In the EDHS-2016, the odds of anaemia were higher among children from households practising open defecation (AOR: 1.49, 95% CI: 1.13–1.90) (Table 5 and Supplementary Files 4).

**Table 5:** Survey-specific adjusted association between anaemia and environmental factors among children 6–59 months in Ethiopia (EDHS-2005, *n* = 4,259; EDHS-2011, *n* = 9,259; EDHS-2016, *n* = 8,399)

Variables	EDHS-2005		EDHS-2011		EDHS-2016	
	**Model 1**	**Model 5**	**Model 1**	**Model 5**	**Model 1**	**Model 2**^**#**^
	**AOR(95%CI)**	**AOR (95%)**	**AOR(95%CI)**	**AOR (95%)**	**AOR(95%CI)**	**AOR (95%)**
***Environmental factors***						
**Sanitation facility**						
Improved	Ref	Ref	Ref	Ref	Ref	Ref
Unimproved	1.18 (0.88–1.57)	0.97 (0.71–1.34)	1.08 (0.92–1.28)	1.02 (0.85–1.21)	0.88 (0.73–1.07)	0.89 (0.69–1.14)
Open defecation	1.26 (0.95–1.66)**	0.93 (0.67–1.28)	1.49 (1.28–1.76)**	1.33 (1.12–1.58)*	1.29 (1.06–1.57)*	1.49 (1.13–1.90)*
**Source of drinking water**						
Improved	Ref	Ref	Ref	Ref	Ref	
Unimproved	0.90 (0.75–1.07)	0.87 (0.72–1.05)	1.17 (1.04–1.32)*	1.11 (0.97–1.26)	1.02 (0.89–1.17)	
**Time to get a water source**						
On-premise	Ref	Ref	Ref	Ref	Ref	
≤ 30 min	1.56 (1.13–2.16)	1.32 (0.91–1.93)	0.99 (0.81–1.23)	0.93 (0.74–1.17)	1.02 (0.83–1.27)	
31–60 min	1.63 (1.14–2.32)	1.44 (0.96–2.17)	1.05 (0.84–1.32)	0.95 (0.74–1.22)	1.10 (0.87–1.39)	
> 60 min	1.81 (1.26–2.61)	1.49 (0.98–2.27)	1.29 (1.02–1.62)*	1.11 (0.85–1.43)	1.01 (0.78–1.29)	
**Housing status**						
Built from finished materials	Ref	Ref	Ref		Ref	
Built from natural or unfinished materials	1.59 (0.94–2.69)	1.37 (0.77–2.45)	1.28 (0.97–1.68)		0.96 (0.74–1.25)	
**Type of cooking fuel**						
Clean fuels	Ref		Ref		Ref	Ref
Solid fuels	1.81 (0.61–5.35)		1.29 (0.71–2.36)		1.62 (1.21–2.18)*	1.44 (0.99–2.11)

*AOR* Adjusted odds ratio

^**^ *p* < 0.001

^*^p < 0.05; Model 1: Environmental factors (from model 0 with *p* < 0.25); Model 5: Environmental factors (from model 4 with *p* < 0.25) + Child related factors (from model 4 with *p* < 0.25) + Maternal factors (from model 4 with *p* < 0.25) + Household factors (from model 4 with *p* < 0.25) + Community level factors (from model 0 with *p* < 0.25); # Model 2 was selected after comparing the log-likelihood values of competing models (see supplementary Table 4)

### Multicollinearity

Multi-collinearity amongst the independent explanatory variables was tested using the Variance Inflation Factor (VIF). It is important to note that most of the literature suggests that VIF values exceeding 5 might indicate a potential collinearity issue. However, our analysis reveals that the independent variables utilized in all models do not exhibit significant collinearity concerns. In the current study, the mean VIF value was estimated to be 1.20 which indicated the absence of multi-collinearity in the models.

## Discussion

### A call for a global effort to address anaemia and to reduce its impact on children

Anaemia is a widespread scourge with serious health implications for children's cognitive, psychological and physical development [51]. To the best of the authors’ knowledge, studies that capture the contributions of environmental factors to anaemia were unavailable in Ethiopia, where one in three households have no toilet facilities and use unimproved water sources. Ninety-three per cent of households use some solid fuel for cooking [21]. The prevalence of childhood anaemia was a significant public health problem affecting one in every child in Ethiopia, and open defecation was a significant key driver for this scourge. We also found that young children, those who had not received deworming medications in the last 6 months, those who were not fully vaccinated, those with mothers who were not working, and those living in poverty were at the highest risk of anaemia. The current study found that 49.3% of children aged 6–59 months had anaemia, a threshold above 40% that the WHO’s classifies as a major public health issue [52]. Exemplified by survey-specific prevalence data surpassing the WHO threshold across years 2005 (47.6%), 2011 (42.8%), and 2016 (57.4%), the anaemia problem in Ethiopia is a protracted public health problem that needs significant effort to address. The most recent EDHS-2016 study revealed a rise in anaemia prevalence of more than 14%, which is worrisome and requires a prompt response. According to Hasan et al. projections, many LIMCs, including Ethiopia, are unlikely to eradicate child anaemia by 2030 [53]. While the current study highlights the need to address anaemia in Ethiopia, this prevalence is not dissimilar to findings elsewhere, especially in developing settings where anaemia remains a significant public health issue. For example, anaemia is a public health problem in countries including India 56% [54], Nepal 52.2%, [55], Bangladesh 52.1% [56] and several SSA countries—Burkina Faso (88%), Mali (82%), Niger (73%), Benin (72%), and Liberia (71%) [57]. This indicates that much more effort and resources are needed to address anaemia as a global scourge and to improve the health outcomes of affected children and individuals. As in many SSA nations including Ethiopia, the high incidence of anaemia may also be related to other factors such as poor child feeding practices, poor dietary diversity, iron deficiencies, and high rates of geohelminth infection in children [3, 4, 7, 10, 37, 58]. In Ethiopia, mothers frequently give children cattle, camel, and goat milk, all of which are known to limit the absorption of iron [59]. More importantly, the higher occurrence of childhood anaemia seems to be linked to the current status of poor sanitation facilities in the country [36], as evidenced in this study.

### Open defecation, geophagia, environmental enteropathy and anaemia in children

In multilevel multivariable pooled models, children from households practising open defecation had increased odds of being anaemic. Our findings are consistent with previous population-based studies, which have reported the association between unimproved household sanitation and higher odds of childhood anaemia [34–36, 60]. Ours, however, is the first population-based study to link open defecation to childhood anaemia in Ethiopia empirically. The link between anaemia and poor sanitation has many plausible explanations, including those associated with intestinal parasite infections and environmental enteropathy [61]. Open defecation spreads worm infections such as hookworm and whipworm infections, both of which are known to induce anaemia through blood loss in the stool, suppression of appetite in children, and competition between the worms and children's nutrient needs [62–65]. In the Ethiopian context, family open-defecation rates have historically been high. However, the trend of family open defecation has shown improvements across EDHS survey periods, reporting including about 61.9% of families defecated in the open in the EDHS-2005, 38% in the EDHS-2011, and 32% in the EDHS-2016 [21, 41, 42]. Despite the decrease in rates of open defecation, children are continuously infected by intestinal helminths due to frequent exposure to unhygienic environments, leading to the loss of iron and iron deficiency anaemia [66]. More evidence of this parasite-related anaemia can be drawn from a systematic review and meta-analysis conducted in Ethiopia, which demonstrated that intestinal parasite infections are highly prevalent (48%) and well distributed across the regional states of Ethiopia [67].

Open defecation, the major discourse in this paper, causes environmental enteropathy, an intestinal inflammation without overt diarrhoea, and occurs in individuals exposed over time to enteric infections resulting from poor sanitation and hygiene [61]. Environmental enteropathy is an important mediator in the relationship between open defecation and nutritional deficits [68]. In many settings in developing countries, children commonly put enteric infected dirt in their mouths (geophagia), exposing themselves to enteric pathogens through fecal–oral exposure [69]. Environmental enteropathy in children causes consistent and chronic intestinal inflammation resulting in micronutrient deficiencies due to poor permeability and malabsorption of essential nutrients. Deficits in the absorption of essential nutrients (both micro- and macronutrients) arising from this loss of surface area could result in a reduction in iron absorption, leading to anaemia [61, 68]. Ferritin, a protein that stores iron inside the red blood cells, is an important factor that supports the transportation of iron in the body and cells [70, 71]. The direct relationship between geophagia and lower ferritin is well established, with anaemia being more common in children suffering from geophagia [4, 72]. There is scarce research and evidence on the effect of environmental enteropathy and geophagia on childhood anaemia in Ethiopia. However, few pocket studies showed a significant association between environmental enteropathy and anaemia [73]. Additionally, the current study indicated that children who had not taken intestinal parasite deworming medicine in the previous 6 months were also at a higher risk of anaemia. This finding was consistent with the Bauleni et al. finding [74], which examined the effects of deworming medication on anaemia among children aged 6–59 months in 17 SSA; showed that children who did not receive deworming medication had increased odds of being anaemic [74]. This may be due to the fact that intestinal parasites can cause anaemia [75], and treating intestinal parasites with deworming medication can reduce the risk of childhood anaemia. It would therefore be plausible to state an indirect contribution of this study to this thesis, given the observed widespread open defecation (enteric pathogens’ soil contaminant) and its relationship with anaemia, assuming that the anaemia could have been a result of environmental enteropathy due to children’s geophagic behaviours.

Our findings have two key implications for researchers and policy makers interested in anemia. Firstly, with respect to research, our study demonstrates the need for future work on anemia and environmental factors. Secondly, a related point about policy is that rural community-level sanitation especially among poor households is important in determining net nutrition because it can cause among infections, intestinal parasitic infections that lead to nutritional loss and malabsorption of essential nutrients (via enteropathy). Therefore, policy actions should be taken at the community level to empower rural communities to take ownership of their sanitation and hygiene facilities, leading to sustainable behavior change and improved child health outcomes especially those among children aged 6–35 months. Furthermore, to combat the significant challenges related to open defecation and anemia, effective policies should focus on the key areas, such as behavior change and community engagement. In this regard, the current ongoing WASH related initiatives of OWNP [16], Co-WASH [17], and the Global WASH program in Ethiopia [18] should be strengthened as they play a pivotal role in ending open defecation and combating anemia in Ethiopia.

### Water sources, solid biofuel, nutrition, children’s age and anaemia in children

Across studies and settings, the relationship between the type of water source and anaemia has been equivocal. The current study findings indicate that an unimproved source of drinking water was not significantly associated with anaemia, which aligns with a pooled study finding from 21 surveys that reported no association between improved household water and anaemia [35]. A study by Kothari et al. found mixed findings on the associations between the water source and anaemia in the analysis of 47 demographic and health surveys [76]. However, only some studies conducted in Zimbabwe [77] and Liberia [78] found a significant association between the use of unimproved sources of drinking water and anaemia in children. These conflicting findings may result from various infectious disease pathogens in various environments, dietary patterns, and other behavioural factors related to water handling and treatment that may influence the relationship between anaemia and the household water source. Further research is warranted to explore and establish the association between the water source and the risk of childhood anaemia.

With regards to the use of solid biofuel, evidence exists indicating that the use of solid biofuel poses a higher risk of anaemia and many adverse effects on child health [79, 80]. In the EDHS-2016 (model 5), the likelihood of anaemia was higher among children from households that used solid fuels. Although the mechanism is puzzling and complex, biofuel smoke contains significant amounts of carbon monoxide, which binds to haemoglobin to generate carboxyhaemoglobin and lowers the amount of haemoglobin in the blood, leading to anaemia [80]. The consistent use of solid biofuel over time may be one of the additional mechanisms that cause anaemia in children in these settings.

We also found that young children (aged between 6–11 months and 12–35 months) were likely to be at the highest risk of anaemia. This is likely due to a complex interplay of factors, including the lack of high-quality iron-rich diets in younger age groups who continue to be breastfed but weaned with diets with poor nutrition appropriate for children. For example, mothers in Ethiopia often give infants cattle milk, which is known to limit the absorption of iron [59]. In addition and due to geophagia, infants are more likely to contract diarrheal infections, which may hinder their ability to ingest and absorb iron, possibly explaining the higher prevalence of anaemia among younger children [21]. These findings were consistent with studies conducted in Ghana [81] and Bangladesh [82] and additional findings where children were reported to be at the highest risk of anaemia in 27 SSA countries [6]. Recognising the risk of anaemia in younger age groups, the WHO recommends daily iron supplementation for all children aged 6 months and older living in areas where anaemia is common [83].

### The setting, socioeconomic determinants and anaemia in children

In the settings where enteric pathogens are endemic, particularly due to open defecation, as discussed elsewhere in this paper, we found that poor households compared to rich households were significantly associated with higher odds of anaemia in children. The finding was somewhat expected as children from poor households may have families who do not have latrines and do open defecation, and may not have access to foods high in iron, vitamin B12, and folic acid, given that poverty is usually linked to food insecurity, leading to increases the risk of developing anaemia. On the other hand, increased wealth increases food availability, access, and diversity in households, lowering the risk of anaemia in children [84]. The connections between household wealth and anaemia identified in this study further imply that socioeconomic factors directly influence children's haemoglobin levels. This finding is consistent with previous related studies in LMICs [6, 8, 82, 85]. Anaemia was more likely to occur among rural residents compared to urban children. A considerable gap in anaemia prevalence between urban and rural areas was reported in several studies [86, 87]. This could be justified by the fact that children in rural Ethiopia have insufficient access to health facilities, lack adequate nutrition, low serving of iron-rich foods, and exposure to environmental contamination. These factors are likely to interact to increase the risk of anaemia in rural children. Anaemia was higher among children born to mothers without formal education than their counterparts. Several studies have shown that low maternal educational status is strongly tied to higher odds of anaemia [6, 8, 88]. This finding is not unexpected, given that low education is strongly linked to child malnutrition [89]. These findings are in line with the recognition of the impact of social factors on health outcomes as recognised by the WHO Social Determinants of Health (SDH) framework [90].

### Study limitations

Although this study contributes to the scant literature on the association between environmental factors and anaemia in Ethiopia, it has limitations. Firstly, it is noteworthy that the home environment is complex, and many other unmeasured environmental factors might be associated with childhood anaemia. Secondly, this study lacks data on child or caregiver hygiene practices, which may be associated with anaemia. Thirdly, data on important pathogens, such as hookworm infections and malaria which are known predictors of anaemia, were also unavailable in EDHS. Fourthly, the quality of water at the home level was not recorded, limiting our ability to properly comprehend the influence of water access on anaemia. Fifthly, since the study was based on secondary data sources, some key variables that may be relevant to anaemia in children, such as household food security, eating habits, level of food availability, access, or diversity, were not adjusted for in our analysis. Sixthly, some variables included in this study were subject to social desirability and recall bias. Seventhly, childhood anaemia testing was limited to Haemoglobin (Hb); no further information on types of anaemia is available in EDHS data. Because this study used EDHS data which lacks variables that would have measured plasma or serum retinol concentration to indicate Vitamin A deficiency, the level of vitamin A during the last 6 months was used as proxy variable. This may have influenced our findings. Finally, since the EDHS was a cross-sectional design, we cannot establish a causal relationship between anaemia and the identified environmental factors. Further, longitudinal studies and controlled trials are needed to established the causal relationships between anaemia and environmental factors reported in this study.

## Conclusion

Anaemia is a serious public health issue in Ethiopia; one in every two children suffers from anaemia. Our study found that open defection was strongly associated with childhood anaemia and open defecation was the main contributor to childhood anaemia in the country. Therefore, to combat the significant challenges related to open defecation and anemia among Ethiopian children, implementing and strengthening effective policies that address the immediate and underlying causes of these key problems is crucial. Comprehensive action, such as access to WASH infrastructure development, behavior change initiatives, community engagement, nutrition interventions, and supplementation programs (iron supplements and deworming), should be prioritized to achieve sustainable improvements. Additionally, the current findings emphasise the need to invest in improving sanitation and ending the practice of open defecation in Ethiopia, which will also have further benefits for addressing other childhood health problems. Since the underlying causes of anaemia are complex, promoting policies, practices and research in integrated approaches, including improving Water, Sanitation, and Hygiene (WASH), iron supplements, deworming, and malaria control, are recommended.

### Supplementary Information

**Additional file 1: Supplementary File 1.** Adjusted association between anaemia and environmental factors and other study covariates among children 6-59 months in Ethiopia, EDHS 2005-2016 (*n*= 21,918).

**Additional file 2: Supplementary File 2.** Adjusted association between anaemia and environmental factors and other study covariates among children 6-59 months in Ethiopia, EDHS 2005 (*n*=4,259).

**Additional file 3: Supplementary File 3.** Adjusted association between anaemia and environmental factors and other study covariates among children 6-59 months in Ethiopia, EDHS 2011 (*n*=9,259).

**Additional file 4: Supplementary File 4.** Adjusted association between anaemia and environmental factors and other study covariates among children 6-59 months in Ethiopia, EDHS 2016 (*n*=8,399).

## Data Availability

Data are available in a public, open access repository in the Measure DHS website (https://www.dhsprogram.com/). Authors requested for the EDHS dataset and obtained permission to access and download the data files for research purpose only after formal online registration and submission of the project title and detail project description. Further information about EDHS data usage and ethical standards are available at: http://dhsprogram.com/data/available-datasets.cfm.
