# Supplementary material for: Household environment associated with anaemia among children aged 6–59 months in Ethiopia: a multilevel analysis of Ethiopia demographic and health survey (2005–2016)
Source: BMC Public Health. 2024 Jan 29;24:315. doi: 10.1186/s12889-024-17780-y (PMC10823679; doi:10.1186/s12889-024-17780-y)
Supplement: Supplementary file 1 — Additional file 1: Supplementary File 1. Adjusted association between anaemia and environmental factors and other study covariates among children 6-59 months in Ethiopia, EDHS 2005-2016 (n= 21,918). [file 12889_2024_17780_MOESM1_ESM.docx]

**Supplementary File 1: Adjusted association between anaemia and environmental factors and other study covariates among children 6-59 months in Ethiopia, EDHS 2005-2016 (n= 21,918)**

| **Variables** | **Model 0** | **Model 1** | | **Model 2** | | **Model 3** | | **Model 4** | | **Model 5** | |
| --- | --- | --- | --- | --- | --- | --- | --- | --- | --- | --- | --- |
|  | **(Null model)** | **AOR(95%CI)** | **p-value** | **AOR (95%CI)** | **p-value** | **AOR (95%CI)** | **p-value** | **AOR (95%CI)** | **p-value** | **AOR (95%CI)** | **p-value** |
| ***Environmental factors*** |  |  |  |  |  |  |  |  |  |  |  |
| **Sanitation facility** |  |  |  |  |  |  |  |  |  |  |  |
| Improved |  | Ref. |  | Ref. |  | Ref. |  | Ref. |  | Ref. |  |
| Unimproved |  | 1.05 (0.94-1.16) | 0.389 | 1.03 (0.91-1.16) | 0.587 | 0.98 (0.87-1.11) | 0.784 | 0.98 (0.87-1.11) | 0.826 | 0.93 (0.82-1.05) | 0.245 |
| Open defecation |  | 1.41 (1.27-1.56)** | p<0.001 | 1.43 (1.27-1.60)** | p<0.001 | 1.29 (1.15-1.46) | p<0.001 | 1.21 (1.06-1.36)* | 0.003 | 1.19 (1.05-1.36) | 0.006 |
| **Source of drinking water** |  |  |  |  |  |  |  |  |  |  |  |
| Improved |  | Ref. |  | Ref. |  | Ref. |  | Ref. |  | Ref. |  |
| Unimproved |  | 1.17 (1.09-1.24)** | p<0.001 | 1.16 (1.08-1.25)** | p<0.001 | 1.13 (1.05-1.22)* | 0.001 | 1.09 (1.01-1.18) | 0.028 | 1.03 (0.95-1.12) | 0.450 |
| **Time to get a water source** |  |  |  |  |  |  |  |  |  |  |  |
| On-premise |  | Ref. |  | Ref. |  | Ref. |  | Ref. |  | Ref. |  |
| ≤ 30 min |  | 0.91 (0.81-1.02) | 0.106 | 0.94 (0.82-1.08) | 0.392 | 0.86 (0.75-0.98)* | 0.035 | 0.85 (0.74-0.98)* | 0.026 | 0.98 (0.85-1.13) | 0.854 |
| 31-60 min |  | 0.99 (0.87-1.13) | 0.969 | 1.04 (0.89-1.21) | 0.565 | 0.95 (0.81-1.11) | 0.502 | 0.94 (0.81-1.09) | 0.434 | 1.04 (0.88-1.23) | 0.602 |
| >60 min |  | 1.14 (0.99-1.30) | 0.052 | 1.23 (1.05-1.44)* | 0.008 | 1.09 (0.93-1.29) | 0.252 | 1.08 (0.93-1.26) | 0.337 | 1.17 (0.98-1.38) | 0.066 |
| **Housing status** |  |  |  |  |  |  |  |  |  |  |  |
| Built from finished materials |  | Ref. |  | Ref. |  | Ref. |  |  |  |  |  |
| Built from natural or unfinished materials |  | 1.11 (0.95-1.29) | 0.183 | 1.18 (0.98-1.42) | 0.071 | 1.10 (0.91-1.33) | 0.307 |  |  |  |  |
| **Type of cooking fuel** |  |  |  |  |  |  |  |  |  |  |  |
| Clean fuels |  | Ref. |  |  |  |  |  |  |  |  |  |
| Solid fuels |  | 1.06 (0.85-1.32) | 0.620 |  |  |  |  |  |  |  |  |
| ***Child factors*** |  |  |  |  |  |  |  |  |  |  |  |
| **Sex** |  |  |  |  |  |  |  |  |  |  |  |
| Male |  |  |  | Ref. |  | Ref. |  | Ref. |  | Ref. |  |
| Female |  |  |  | 0.95 (0.89-1.02) | 0.144 | 0.95 (0.89-1.02) | 0.187 | 0.96 (0.89-1.02) | 0.210 | 0.96 (0.89-1.02) | 0.214 |
| **Age (months)** |  |  |  |  |  |  |  |  |  |  |  |
| 6-11 |  |  |  | 3.62 (3.23-4.06)** | p<0.001 | 3.63 (3.25-4.07) | p<0.001 | 3.61 (3.22-4.03)** | p<0.001 | 2.94 (2.62-3.30)** | p<0.001 |
| 12-23 |  |  |  | 3.38 (3.08-3.70)** | p<0.001 | 3.38 (3.09-3.69) | p<0.001 | 3.39 (3.10-3.71)** | p<0.001 | 2.70 (2.46-2.97)** | p<0.001 |
| 24-35 |  |  |  | 2.07 (1.90-2.26)** | p<0.001 | 2.06 (1.89-2.25) | p<0.001 | 2.06 (1.89-2.24) | p<0.001 | 1.62 (1.48-1.77)** | p<0.001 |
| 36-59 |  |  |  | Ref. |  | Ref. |  | Ref. |  | Ref. |  |
| **Size of the child at birth** |  |  |  |  |  |  |  |  |  |  |  |
| Larger |  |  |  | Ref. |  | Ref. |  |  |  |  |  |
| Average |  |  |  | 0.99 (0.91-1.07) | 0.790 | 0.97 (0.89-1.05) | 0.449 |  |  |  |  |
| Small |  |  |  | 1.06 (0.97-1.15) | 0.217 | 1.03 (0.94-1.13) | 0.482 |  |  |  |  |
| **Full vaccination** |  |  |  |  |  |  |  |  |  |  |  |
| Yes |  |  |  | Ref. |  | Ref. |  | Ref. |  | Ref. |  |
| No |  |  |  | 1.12 (1.03-1.22)* | 0.006 | 1.09 (1.01-1.18)* | 0.042 | 1.08 (1.00-1.18)* | 0.049 | 1.14 (1.05-1.24)* | 0.002 |
| **Received deworming medication in the last 6 months** |  |  |  |  |  |  |  |  |  |  |  |
| Yes |  |  |  | Ref. |  | Ref. |  | Ref. |  | Ref. |  |
| No |  |  |  | 1.12 (1.01-1.25)* | 0.027 | 1.10 (0.99-1.22) | 0.069 | 1.11 (0.99-1.23) | 0.057 | 1.11 (1.01-1.24)* | 0.048 |
| **Iron supplementation** |  |  |  |  |  |  |  |  |  |  |  |
| Yes |  |  |  | 0.88 (0.77-1.02) | 0.086 | 0.90 (0.78-1.04) | 0.158 | 0.91 (0.78-1.04) | 0.178 | 0.95 (0.82-1.09) | 0.496 |
| No |  |  |  | Ref. |  | Ref. |  | Ref. |  | Ref. |  |
| **Vitamin A last 6 months** |  |  |  |  |  |  |  |  |  |  |  |
| Yes |  |  |  | Ref. |  | Ref. |  | Ref. |  | Ref. |  |
| No |  |  |  | 1.09 (1.02-1.18)* | 0.011 | 1.06 (0.99-1.14) | 0.090 | 1.06 (0.98-1.14) | 0.098 | 1.02 (0.94-1.09) | 0.673 |
| **Currently breastfeeding** |  |  |  |  |  |  |  |  |  |  |  |
| Yes |  |  |  | Ref. |  |  |  |  |  |  |  |
| No |  |  |  | 1.01 (0.93-1.08) | 0.978 |  |  |  |  |  |  |
| **Birth interval** |  |  |  |  |  |  |  |  |  |  |  |
| 7- 33 months |  |  |  | Ref. |  |  |  |  |  |  |  |
| ≥ 33 months |  |  |  | 0.99 (0.92-1.06) | 0.782 |  |  |  |  |  |  |
| **Diarrhoea** |  |  |  |  |  |  |  |  |  |  |  |
| Yes |  |  |  | 1.05 (0.95-1.15) | 0.291 |  |  |  |  |  |  |
| No |  |  |  | Ref. |  |  |  |  |  |  |  |
| ***Parental factors*** |  |  |  |  |  |  |  |  |  |  |  |
| **Mother's age** |  |  |  |  |  |  |  |  |  |  |  |
| 15-18 |  |  |  |  |  | 0.73 (0.47-1.14) | 0.168 | 0.73 (0.47-1.14) | 0.167 | 0.71 (0.45-1.11) | 0.136 |
| 18-24 |  |  |  |  |  | 1.04 (0.94-1.15) | 0.451 | 1.04 (0.94-1.14) | 0.476 | 1.01 (0.91-1.11) | 0.905 |
| 25-34 |  |  |  |  |  | 1.05 (0.97-1.14) | 0.214 | 1.05 (0.97-1.15) | 0.203 | 1.03 (0.94-1.12) | 0.514 |
| 35-49 |  |  |  |  |  | Ref. |  | Ref. |  | Ref. |  |
| **Mother's education** |  |  |  |  |  |  |  |  |  |  |  |
| No education |  |  |  |  |  | 1.04 (0.96-1.14) | 0.278 |  |  |  |  |
| Primary and above |  |  |  |  |  | Ref. |  |  |  |  |  |
| **Mother's currently working.** |  |  |  |  |  |  |  |  |  |  |  |
| Yes |  |  |  |  |  | Ref. |  | Ref. |  | Ref. |  |
| No |  |  |  |  |  | 1.11 (1.03-1.20)* | 0.007 | 1.11 (1.03-1.19)* | 0.007 | 1.10 (1.02-1.19)* | 0.013 |
| **Maternal BMI (kg/m^2^)** |  |  |  |  |  |  |  |  |  |  |  |
| <18.5 |  |  |  |  |  | Ref. |  | Ref. |  | Ref. |  |
| 18.5 to 24.9 |  |  |  |  |  | 0.84 (0.77-0.90)** | p<0.001 | 0.85 (0.78-0.91) | p<0.001 | 0.84 (0.78-0.91)** | p<0.001 |
| 25 + |  |  |  |  |  | 0.84 (0.73-1.03) | 0.054 | 0.86 (0.73-1.01) | 0.066 | 0.82 (0.69-0.96)* | 0.014 |
| **Listening to radio** |  |  |  |  |  |  |  |  |  |  |  |
| Yes |  |  |  |  |  | Ref. |  | Ref. |  | Ref. |  |
| Not at all |  |  |  |  |  | 1.12 (1.04-1.21)* | 0.004 | 1.08 (1.01-1.18)* | 0.041 | 1.04 (0.95-1.12) | 0.370 |
| **Watching television** |  |  |  |  |  |  |  |  |  |  |  |
| Yes |  |  |  |  |  | Ref. |  | Ref. |  | Ref. |  |
| Not at all |  |  |  |  |  | 1.27 (1.15-1.41)** | p<0.001 | 1.26 (1.14-1.39)** | p<0.001 | 1.27 (1.14-1.40)** | p<0.001 |
| ***Household factors*** |  |  |  |  |  |  |  |  |  |  |  |
| **Wealth index** |  |  |  |  |  |  |  |  |  |  |  |
| Poor |  |  |  |  |  |  |  | 1.27 (1.15-1.40)** | p<0.001 | 1.18 (1.06-1.31)* | 0.002 |
| Middle |  |  |  |  |  |  |  | 1.05 (0.94-1.17) | 0.354 | 1.01 (0.90-1.13) | 0.811 |
| Rich |  |  |  |  |  |  |  | Ref. |  | Ref. |  |
| ***Community-level characteristics*** |  |  |  |  |  |  |  |  |  |  |  |
| **Residence** |  |  |  |  |  |  |  |  |  |  |  |
| Urban |  |  |  |  |  |  |  |  |  | Ref. |  |
| Rural |  |  |  |  |  |  |  |  |  | 1.23 (1.06-1.42)* | 0.005 |
| **Region** |  |  |  |  |  |  |  |  |  |  |  |
| Agrarian |  |  |  |  |  |  |  |  |  | Ref. |  |
| Pastoralist |  |  |  |  |  |  |  |  |  | 1.58 (1.44-1.74)** | p<0.001 |
| City administration |  |  |  |  |  |  |  |  |  | 1.43 (1.27-1.62)** | p<0.001 |
| **EDHS** |  |  |  |  |  |  |  |  |  |  |  |
| 2005 |  |  |  |  |  |  |  |  |  | 0.45 (0.40-0.51)** | p<0.001 |
| 2011 |  |  |  |  |  |  |  |  |  | 0.50 (0.46-0.55)** | p<0.001 |
| 2016 |  |  |  |  |  |  |  |  |  | Ref. |  |
| **Random effect** |  |  |  |  |  |  |  |  |  |  |  |
| ICC (%) | 6.38 | 5.48 |  | 5.02 |  | 4.86 |  | 4.93 |  | 4.24 |  |
| Log-likelihood | -14060.391 | -137,99.074 |  | -10389.692 |  | -10325.692 |  | -10360.817 |  | -10177.156 |  |

AOR (Adjusted Odds Ratio); LL: Log-likelihood; *p-value<0.05; **p<0.001

Model 0: Empty model with no independent variables

Model 1: All environmental factors were included in the model

Model 2: Environmental factors (from model 1 with p<0.25) + Child-related factors (from model 0 with p<0.25)

Model 3: Environmental factors (from model 2 with p<0.25) + Child-related factors (from model 2 with p<0.25) + Maternal factors (from model 0 with p<0.25)

Model 4: Environmental factors (from model 3 with p<0.25) + Child-related factors (from model 3 with p<0.25) + Maternal factors (from model 3 with p<0.25)+ Household factors (from model 0 with p<0.25)

Model 5: Environmental factors (from model 4 with p<0.25) +Child related factors (from model 4 with p<0.25) + Maternal factors (from model 4 with p<0.25)+ Household factors (from model 4 with p<0.25) + Community level factors (from model 0 with p<0.25)
