# Supplementary material for: Household environment associated with anaemia among children aged 6–59 months in Ethiopia: a multilevel analysis of Ethiopia demographic and health survey (2005–2016)
Source: BMC Public Health. 2024 Jan 29;24:315. doi: 10.1186/s12889-024-17780-y (PMC10823679; doi:10.1186/s12889-024-17780-y)
Supplement: Supplementary file 2 — Additional file 2: Supplementary File 2. Adjusted association between anaemia and environmental factors and other study covariates among children 6-59 months in Ethiopia, EDHS 2005 (n=4,259). [file 12889_2024_17780_MOESM2_ESM.docx]

**Supplementary File 2: Adjusted association between anaemia and environmental factors and other study covariates among children 6-59 months in Ethiopia, EDHS 2005 (n=4,259)**

| **Variables** | **Model 0** | **Model 1** | | **Model 2** | | **Model 3** | | **Model 4** | | **Model 5** | |
| --- | --- | --- | --- | --- | --- | --- | --- | --- | --- | --- | --- |
|  | **Null model** | **AOR(95%CI)** | **p-value** | **AOR (95%CI)** | **p-value** | **AOR (95%CI)** | **p-value** | **AOR (95%CI)** | **p-value** | **AOR (95%)** | **p-value** |
| ***Environmental factors*** |  |  |  |  |  |  |  |  |  |  |  |
| **Sanitation facility** |  |  |  |  |  |  |  |  |  |  |  |
| Improved |  | Ref. |  | Ref. |  | Ref. |  | Ref. |  | Ref. |  |
| Unimproved |  | 1.18 (0.88-1.57) | 0.264 | 1.08 (0.79-1.46) | 0.634 | 1.02 (0.74-1.38) | 0.900 | 1.01 (0.74-1.37) | 0.967 | 0.97 (0.71-1.34) | 0.878 |
| Open defecation |  | 1.26 (0.95-1.66) | 0.096 | 1.25 (0.93-1.68) | 0.131 | 1.10 (0.82-1.49) | 0.519 | 1.01 (0.73-1.38) | 0.969 | 0.93 (0.67-1.28) | 0.652 |
| **Source of drinking water** |  |  |  |  |  |  |  |  |  |  |  |
| Improved |  | Ref. |  | Ref. |  | Ref. |  | Ref. |  | Ref. |  |
| Unimproved |  | 0.90 (0.75-1.07) | 0.251 | 0.86 (0.71-1.04) | 0.120 | 0.88 (0.73-1.06) | 0.173 | 0.87 (0.72-1.05) | 0.155 | 0.87 (0.72-1.05) | 0.154 |
| **Time to get a water source** |  |  |  |  |  |  |  |  |  |  |  |
| On-premise |  | Ref. |  | Ref. |  | Ref. |  | Ref. |  | Ref. |  |
| ≤ 30 min |  | 1.56 (1.13-2.16)* | 0.007 | 1.74 (1.22-2.47)* | 0.002 | 1.50 (1.05-2.15)* | 0.026 | 1.46 (1.02-2.09)* | 0.037 | 1.32 (0.91-1.93) | 0.139 |
| 31-60 min |  | 1.63 (1.14-2.32)* | 0.007 | 1.75 (1.19-2.58)* | 0.004 | 1.65 (1.11-2.44)* | 0.012 | 1.61 (1.09-2.38)* | 0.016 | 1.44 (0.96-2.17) | 0.074 |
| >60 min |  | 1.81 (1.26-2.61)* | 0.001 | 2.05 (1.38-3.06)** | p<0.001 | 1.77 (1.18-2.65)* | 0.005 | 1.69 (1.13-2.53)* | 0.011 | 1.49 (0.98-2.27) | 0.057 |
| **Housing status** |  |  |  |  |  |  |  |  |  |  |  |
| Built from finished materials |  | Ref. |  | Ref. |  | Ref. |  | Ref. |  | Ref. |  |
| Built from natural or unfinished materials |  | 1.59 (0.94-2.69) | 0.085 | 1.72 (0.96-3.07) | 0.068 | 1.46 (0.82-2.59) | 0.193 | 1.41 (0.79-2.50) | 0.235 | 1.37 (0.77-2.45) | 0.277 |
| **Type of cooking fuel** |  |  |  |  |  |  |  |  |  |  |  |
| Clean fuels |  | Ref. |  |  |  |  |  |  |  |  |  |
| Solid fuels |  | 1.81 (0.61-5.35) | 0.283 |  |  |  |  |  |  |  |  |
| ***Child factors*** |  |  |  |  |  |  |  |  |  |  |  |
| **Sex** |  |  |  |  |  |  |  |  |  |  |  |
| Male |  |  |  | Ref. |  | Ref. |  |  |  |  |  |
| Female |  |  |  | 0.89 (0.78-1.04) | 0.148 | 0.94 (0.81-1.08) | 0.367 |  |  |  |  |
| **Age (months)** |  |  |  |  |  |  |  |  |  |  |  |
| 6-11 |  |  |  | 2.23 (1.73-2.86)** | p<0.001 | 2.39 (1.88-3.04)** | p<0.001 | 2.41 (1.90-3.05)** | p<0.001 | 2.41 (1.90-3.06)** | p<0.001 |
| 12-23 |  |  |  | 2.37 (1.95-2.88)** | p<0.001 | 2.59 (2.15-3.13)** | p<0.001 | 2.61 (2.17-3.14)** | p<0.001 | 2.61 (2.17-3.14)** | p<0.001 |
| 24-35 |  |  |  | 1.31 (1.08-1.58)** | p<0.001 | 1.33 (1.10-1.59)** | 0.002 | 1.35 (1.12-1.62)* | 0.001 | 1.35 (1.12-1.62)** | p<0.001 |
| 36-59 |  |  |  | Ref. |  | Ref. |  | Ref. |  | Ref. |  |
| **Birth interval** |  |  |  |  |  |  |  |  |  |  |  |
| 7- 33 months |  |  |  | Ref. |  | Ref. |  | Ref. |  | Ref. |  |
| ≥ 33 months |  |  |  | 1.17 (1.01-1.37)* | 0.045 | 1.24 (0.89-1.72) | 0.204 | 1.09 (0.94-1.28) | 0.225 | 1.09 (0.94-1.27) | 0.247 |
| **Size of the child at birth** |  |  |  |  |  |  |  |  |  |  |  |
| Larger |  |  |  | Ref. |  | Ref. |  | Ref. |  | Ref. |  |
| Average |  |  |  | 0.92 (0.78-1.09) | 0.371 | 0.88 (0.74-1.04) | 0.134 | 0.87 (0.74-1.04) | 0.119 | 0.88 (0.74-1.04) | 0.148 |
| Small |  |  |  | 0.84 (0.69-1.02) | 0.088 | 0.80 (0.67-0.97)* | 0.026 | 0.79 (0.66-0.96)* | 0.017 | 0.80 (0.66-0.96)* | 0.019 |
| **Currently breastfeeding** |  |  |  |  |  |  |  |  |  |  |  |
| Yes |  |  |  | Ref. |  |  |  |  |  |  |  |
| No |  |  |  | 0.90 (0.76-1.08) | 0.277 |  |  |  |  |  |  |
| **Full vaccination** |  |  |  |  |  |  |  |  |  |  |  |
| Yes |  |  |  | Ref. |  |  |  |  |  |  |  |
| No |  |  |  | 1.03 (0.83-1.26) | 0.796 |  |  |  |  |  |  |
| **Vitamin A last 6 months** |  |  |  |  |  |  |  |  |  |  |  |
| Yes |  |  |  | Ref. |  | Ref. |  | Ref. |  | Ref. |  |
| No |  |  |  | 0.89 (0.76-1.04) | 0.138 | 0.87 (0.75-1.02) | 0.079 | 0.87 (0.75-1.01) | 0.073 | 0.85 (0.73-0.99)* | 0.043 |
| **Diarrhoea** |  |  |  |  |  |  |  |  |  |  |  |
| Yes |  |  |  | 1.20 (0.99-1.45) | 0.059 | 1.20 (0.99-1.45) | 0.050 | 1.19 (0.99-1.44) | 0.063 | 1.19 (0.98-1.44) | 0.064 |
| No |  |  |  | Ref. |  | Ref. |  | Ref. |  | Ref. |  |
| ***Parental factors*** |  |  |  |  |  |  |  |  |  |  |  |
| **Mother's age** |  |  |  |  |  |  |  |  |  |  |  |
| 15-18 |  |  |  |  |  | 0.85 (0.27-2.65) | 0.778 |  |  |  |  |
| 18-24 |  |  |  |  |  | 1.19 (0.81-1.75) | 0.378 |  |  |  |  |
| 25-34 |  |  |  |  |  | 1.14 (0.81-1.60) | 0.760 |  |  |  |  |
| 35-49 |  |  |  |  |  | Ref. |  |  |  |  |  |
| **Mother's education** |  |  |  |  |  |  |  |  |  |  |  |
| No education |  |  |  |  |  | 0.95 (0.78-1.17) | 0.665 |  |  |  |  |
| Primary and above |  |  |  |  |  | Ref. |  |  |  |  |  |
| **Mother's currently working.** |  |  |  |  |  |  |  |  |  |  |  |
| Yes |  |  |  |  |  | Ref. |  | Ref. |  | Ref. |  |
| No |  |  |  |  |  | 0.83 (0.70-0.99)* | 0.040 | 0.83 (0.70-0.98)* | 0.036 | 0.82 (0.69-0.97)* | 0.024 |
| **Maternal BMI (kg/m^2^)** |  |  |  |  |  |  |  |  |  |  |  |
| <18.5 |  |  |  |  |  | Ref. |  | Ref. |  | Ref. |  |
| 18.5 to 24.9 |  |  |  |  |  | 0.81 (0.68-0.96)* | 0.018 | 0.82 (0.69-0.97)* | 0.023 | 0.82 (0.69-0.96)* | 0.019 |
| 25 + |  |  |  |  |  | 0.83 (0.55-1.23) | 0.363 | 0.84 (0.57-1.26) | 0.414 | 0.87 (0.58-1.30) | 0.506 |
| **Listening to radio** |  |  |  |  |  |  |  |  |  |  |  |
| Yes |  |  |  |  |  | Ref. |  |  |  |  |  |
| Not at all |  |  |  |  |  | 1.08 (0.90-1.27) | 0.399 |  |  |  |  |
| **Watching television** |  |  |  |  |  |  |  |  |  |  |  |
| Yes |  |  |  |  |  | Ref. |  | Ref. |  | Ref. |  |
| Not at all |  |  |  |  |  | 1.57 (1.15-2.14)* | 0.004 | 1.52 (1.13-2.06)* | 0.006 | 1.36 (0.98-1.88) | 0.060 |
| ***Household factors*** |  |  |  |  |  |  |  |  |  |  |  |
| **Wealth index** |  |  |  |  |  |  |  |  |  |  |  |
| Poor |  |  |  |  |  |  |  | 1.23 (1.01-1.51)* | 0.044 | 1.19 (0.96-1.46) | 0.099 |
| Middle |  |  |  |  |  |  |  | 1.24 (0.99-1.55) | 0.053 | 1.19 (0.95-1.49) | 0.128 |
| Rich |  |  |  |  |  |  |  | Ref. |  | Ref. |  |
| ***Community-level characteristics*** |  |  |  |  |  |  |  |  |  |  |  |
| **Residence** |  |  |  |  |  |  |  |  |  |  |  |
| Urban |  |  |  |  |  |  |  |  |  | Ref. |  |
| Rural |  |  |  |  |  |  |  |  |  | 1.64 (1.15-2.34)* | 0.006 |
| **Region** |  |  |  |  |  |  |  |  |  |  |  |
| Agrarian |  |  |  |  |  |  |  |  |  | Ref. |  |
| Pastoralist |  |  |  |  |  |  |  |  |  | 1.07 (0.87-1.32) | 0.492 |
| City administration |  |  |  |  |  |  |  |  |  | 1.26 (0.93-1.72) | 0.136 |
| **Random effect** |  |  |  |  |  |  |  |  |  |  |  |
| ICC (%) | 8.38 | 6.34 |  | 7.81 |  | 7.61 |  | 7.69 |  | 7.54 |  |
| Log-likelihood | -2654.7561 | -2616.2531 |  | -2293.5563 |  | -2451.6138 |  | -2451.4161 |  | -2447.0034 |  |

AOR (Adjusted Odds Ratio); LL: Log-likelihood; *p-value<0.05; **p<0.001

Model 0: Empty model with no independent variables

Model 1: All environmental factors were included in the model

Model 2: Environmental factors (from model 1 with p<0.25) + Child-related factors (from model 0 with p<0.25)

Model 3: Environmental factors (from model 2 with p<0.25) + Child-related factors (from model 2 with p<0.25) + Maternal factors (from model 0 with p<0.25)

Model 4: Environmental factors (from model 3 with p<0.25) + Child-related factors (from model 3 with p<0.25) + Maternal factors (from model 3 with p<0.25)+ Household factors (from model 0 with p<0.25)

Model 5: Environmental factors (from model 4 with p<0.25) +Child related factors (from model 4 with p<0.25) + Maternal factors (from model 4 with p<0.25)+ Household factors (from model 4 with p<0.25) + Community level factors (from model 0 with p<0.25)
