# Supplementary material for: Household environment associated with anaemia among children aged 6–59 months in Ethiopia: a multilevel analysis of Ethiopia demographic and health survey (2005–2016)
Source: BMC Public Health. 2024 Jan 29;24:315. doi: 10.1186/s12889-024-17780-y (PMC10823679; doi:10.1186/s12889-024-17780-y)
Supplement: Supplementary file 3 — Additional file 3: Supplementary File 3. Adjusted association between anaemia and environmental factors and other study covariates among children 6-59 months in Ethiopia, EDHS 2011 (n=9,259). [file 12889_2024_17780_MOESM3_ESM.docx]

**Supplementary File 3: Adjusted association between anaemia and environmental factors and other study covariates among children 6-59 months in Ethiopia, EDHS 2011 (n=9,259)**

| **Variables** | **Model 0** | **Model 1** | | **Model 2** | | **Model 3** | | **Model 4** | | **Model 5** | |
| --- | --- | --- | --- | --- | --- | --- | --- | --- | --- | --- | --- |
|  | **Null model** | **AOR(95%CI)** | **p-value** | **AOR (95%CI)** | **p-value** | **AOR (95%CI)** | **p-value** | **AOR (95%CI)** | **p-value** | **AOR (95%)** | **p-value** |
| ***Environmental factors*** |  |  |  |  |  |  |  |  |  |  |  |
| **Sanitation facility** |  |  |  |  |  |  |  |  |  |  |  |
| Improved |  | Ref. |  | Ref. |  | Ref. |  | Ref. |  | Ref. |  |
| Unimproved |  | 1.08 (0.92-1.28) | 0.324 | 1.08 (0.91-1.28) | 0.381 | 1.04 (0.87-1.24) | 0.643 | 1.06 (0.89-1.26) | 0.464 | 1.02 (0.85-1.21) | 0.857 |
| Open defecation |  | 1.49 (1.28-1.76)* | p<0.001 | 1.49 (1.26-1.78)** | p<0.001 | 1.41 (1.19-1.68)** | p<0.001 | 1.39 (1.17-1.67)** | p<0.001 | 1.33 (1.12-1.58)* | 0.001 |
| **Source of drinking water** |  |  |  |  |  |  |  |  |  |  |  |
| Improved |  | Ref. |  | Ref. |  | Ref. |  | Ref. |  | Ref. |  |
| Unimproved |  | 1.17 (1.04-1.32)* | 0.010 | 1.16 (1.03-1.32)* | 0.018 | 1.11 (0.98-1.26) | 0.102 | 1.09 (0.96-1.24) | 0.172 | 1.11 (0.97-1.26) | 0.128 |
| **Time to get a water source** |  |  |  |  |  |  |  |  |  |  |  |
| On-premise |  | Ref. |  | Ref. |  | Ref. |  | Ref. |  | Ref. |  |
| ≤ 30 min |  | 0.99 (0.81-1.23) | 0.995 | 1.03 (0.82-1.29) | 0.779 | 0.97 (0.77-1.21) | 0.773 | 0.98 (0.78-1.22) | 0.871 | 0.93 (0.74-1.17) | 0.541 |
| 31-60 min |  | 1.05 (0.84-1.32) | 0.656 | 1.10 (0.87-1.40) | 0.426 | 1.01 (0.79-1.29) | 0.909 | 1.03 (0.81-1.31) | 0.808 | 0.95 (0.74-1.22) | 0.688 |
| >60 min |  | 1.29 (1.02-1.62)* | 0.033 | 1.36 (1.06-1.73)* | 0.014 | 1.24 (0.96-1.59) | 0.088 | 1.25 (0.98-1.60) | 0.069 | 1.11 (0.85-1.43) | 0.429 |
| **Housing status** |  |  |  |  |  |  |  |  |  |  |  |
| Built from finished materials |  | Ref. |  | Ref. |  | Ref. |  |  |  |  |  |
| Built from natural or unfinished materials |  | 1.28 (0.97-1.68) | 0.071 | 1.28 (0.96-1.71) | 0.085 | 1.17 (0.87-1.56) | 0.298 |  |  |  |  |
| **Type of cooking fuel** |  |  |  |  |  |  |  |  |  |  |  |
| Clean fuels |  | Ref. |  |  |  |  |  |  |  |  |  |
| Solid fuels |  | 1.29 (0.71-2.36) | 0.283 |  |  |  |  |  |  |  |  |
| ***Child factors*** |  |  |  |  |  |  |  |  |  |  |  |
| **Sex** |  |  |  |  |  |  |  |  |  |  |  |
| Male |  |  |  | Ref. |  |  |  |  |  |  |  |
| Female |  |  |  | 0.97 (0.88-1.06) | 0.519 |  |  |  |  |  |  |
| **Age (months)** |  |  |  |  |  |  |  |  |  |  |  |
| 6-11 |  |  |  | 2.98 (2.52-3.53)** | p<0.001 | 3.03 (2.56-3.59)** | p<0.001 | 3.01 (2.54-3.56)** | p<0.001 | 3.02 (2.55-3.57)** | p<0.001 |
| 12-23 |  |  |  | 2.79 (2.45-3.19)** | p<0.001 | 2.86 (2.50-3.26)** | p<0.001 | 2.89 (2.53-3.30)** | p<0.001 | 2.87 (2.51-3.28)** | p<0.001 |
| 24-35 |  |  |  | 1.81 (1.60-2.04)** | p<0.001 | 1.82 (1.61-2.06)** | p<0.001 | 1.83 (1.61-2.06)** | p<0.001 | 1.81 (1.60-2.05)** | p<0.001 |
| 36-59 |  |  |  | Ref. |  | Ref. |  | Ref. |  | Ref. |  |
| **Birth interval** |  |  |  |  |  |  |  |  |  |  |  |
| 7- 33 months |  |  |  | Ref. |  |  |  |  |  |  |  |
| ≥ 33 months |  |  |  | 1.01 (0.91-1.12) | 0.839 |  |  |  |  |  |  |
| **Size of the child at birth** |  |  |  |  |  |  |  |  |  |  |  |
| Larger |  |  |  | Ref. |  | Ref. |  |  |  |  |  |
| Average |  |  |  | 0.97 (0.87-1.09) | 0.655 | 0.95 (0.84-1.07) | 0.397 |  |  |  |  |
| Small |  |  |  | 1.08 (0.95-1.22) | 0.213 | 1.05 (0.93-1.19) | 0.447 |  |  |  |  |
| **Currently breastfeeding** |  |  |  |  |  |  |  |  |  |  |  |
| Yes |  |  |  | Ref. |  | Ref. |  | Ref. |  | Ref. |  |
| No |  |  |  | 0.88 (0.79-0.98)* | 0.024 | 0.89 (0.80-0.99)* | 0.050 | 0.89 (0.80-0.99)* | 0.049 | 0.89 (0.79-0.99)* | 0.045 |
| **Full vaccination** |  |  |  |  |  |  |  |  |  |  |  |
| Yes |  |  |  | Ref. |  | Ref. |  | Ref. |  | Ref. |  |
| No |  |  |  | 1.10 (0.98-1.25) | 0.110 | 1.09 (0.97-1.23) | 0.152 | 1.09 (0.97-1.23) | 0.149 | 1.06 (0.93-1.19) | 0.391 |
| **Received deworming medication in the last 6 months** |  |  |  |  |  |  |  |  |  |  |  |
| Yes |  |  |  | Ref. |  | Ref. |  | Ref. |  | Ref. |  |
| No |  |  |  | 1.09 (0.96-1.25) | 0.166 | 1.08 (0.94-1.23) | 0.238 | 1.08 (0.95-1.23) | 0.235 | 1.09 (0.96-1.25) | 0.156 |
| **Iron supplementation** |  |  |  |  |  |  |  |  |  |  |  |
| Yes |  |  |  | Ref. |  | Ref. |  | Ref. |  | Ref. |  |
| No |  |  |  | 0.84 (0.70-1.01) | 0.058 | 0.85 (0.71-1.02) | 0.080 | 0.85 (0.71-1.02) | 0.093 | 0.85 (0.71-1.02) | 0.085 |
| **Vitamin A last 6 months** |  |  |  |  |  |  |  |  |  |  |  |
| Yes |  |  |  | Ref. |  | Ref. |  | Ref. |  | Ref. |  |
| No |  |  |  | 1.15 (1.03-1.28)* | 0.014 | 1.13 (1.01-1.26)* | 0.030 | 1.12 (1.01-1.25)* | 0.034 | 1.11 (0.99-1.23) | 0.062 |
| **Diarrhoea** |  |  |  |  |  |  |  |  |  |  |  |
| Yes |  |  |  | 1.17 (1.02-1.33)* | 0.023 | 1.16 (1.02-1.33)* | 0.027 | 1.18 (1.03-1.35)* | 0.016 | 1.20 (1.05-1.37)* | 0.007 |
| No |  |  |  | Ref. |  | Ref. |  | Ref. |  | Ref. |  |
| ***Parental factors*** |  |  |  |  |  |  |  |  |  |  |  |
| **Mother's age** |  |  |  |  |  |  |  |  |  |  |  |
| 15-18 |  |  |  |  |  | 0.52 (0.27-0.98)* | 0.045 | 0.52 (0.27-0.99)* | 0.047 | 0.52 (0.28-0.99)* | 0.049 |
| 18-24 |  |  |  |  |  | 1.02 (0.88-1.18) | 0.795 | 1.03 (0.89-1.19) | 0.667 | 1.01 (0.86-1.16) | 0.932 |
| 25-34 |  |  |  |  |  | 1.04 (0.92-1.17) | 0.523 | 1.04 (0.92-1.17) | 0.498 | 1.02 (0.91-1.15) | 0.707 |
| 35-49 |  |  |  |  |  | Ref. |  | Ref. |  | Ref. |  |
| **Mother's education** |  |  |  |  |  |  |  |  |  |  |  |
| No education |  |  |  |  |  | 1.14 (1.01-1.29)* | 0.029 | 1.15 (1.02-1.30)* | 0.022 | 1.13 (1.06-1.32)* | 0.003 |
| Primary and above |  |  |  |  |  | Ref. |  | Ref. |  | Ref. |  |
| **Mother's currently working.** |  |  |  |  |  |  |  |  |  |  |  |
| Yes |  |  |  |  |  | Ref. |  | Ref. |  | Ref. |  |
| No |  |  |  |  |  | 1.19 (1.06-1.32)* | 0.002 | 1.18 (1.06-1.32)* | 0.003 | 1.18 (1.06-1.32)* | 0.003 |
| **Maternal BMI (kg/m^2^)** |  |  |  |  |  |  |  |  |  |  |  |
| <18.5 |  |  |  |  |  | Ref. |  | Ref. |  | Ref. |  |
| 18.5 to 24.9 |  |  |  |  |  | 0.89 (0.80-1.00) | 0.055 | 0.91 (0.81-1.01) | 0.083 | 0.90 (0.80-1.01) | 0.064 |
| 25 + |  |  |  |  |  | 0.76 (0.59-0.97)* | 0.028 | 0.75 (0.59-0.96)* | 0.022 | 0.77 (0.61-0.98)* | 0.036 |
| **Listening to radio** |  |  |  |  |  |  |  |  |  |  |  |
| Yes |  |  |  |  |  | Ref. |  |  |  |  |  |
| Not at all |  |  |  |  |  | 0.98 (0.88-1.09) | 0.736 |  |  |  |  |
| **Watching television** |  |  |  |  |  |  |  |  |  |  |  |
| Yes |  |  |  |  |  | Ref. |  | Ref. |  | Ref. |  |
| Not at all |  |  |  |  |  | 1.18 (1.04-1.34)* | 0.012 | 1.17 (1.04-1.33)* | 0.011 | 1.19 (1.06-1.35)* | 0.005 |
| ***Household factors*** |  |  |  |  |  |  |  |  |  |  |  |
| **Wealth index** |  |  |  |  |  |  |  |  |  |  |  |
| Poor |  |  |  |  |  |  |  | 1.08 (0.93-1.26) | 0.306 |  |  |
| Middle |  |  |  |  |  |  |  | 0.96 (0.81-1.12) | 0.600 |  |  |
| Rich |  |  |  |  |  |  |  | Ref. |  |  |  |
| ***Community-level characteristics*** |  |  |  |  |  |  |  |  |  |  |  |
| **Residence** |  |  |  |  |  |  |  |  |  |  |  |
| Urban |  |  |  |  |  |  |  |  |  | Ref. |  |
| Rural |  |  |  |  |  |  |  |  |  | 1.34 (1.06-1.69)* | 0.015 |
| **Region** |  |  |  |  |  |  |  |  |  |  |  |
| Agrarian |  |  |  |  |  |  |  |  |  | Ref. |  |
| Pastoralist |  |  |  |  |  |  |  |  |  | 2.04 (1.72-2.43)* | p<0.001 |
| City administration |  |  |  |  |  |  |  |  |  | 1.40 (1.13-1.74)* | 0.002 |
| **Random effect** |  |  |  |  |  |  |  |  |  |  |  |
| ICC (%) | 13.68 | 10.43 |  | 11.47 |  | 10.9 |  | 11.08 |  | 9.22 |  |
| Log-likelihood | -6030.0278 | -5889.940 |  | -5427.1888 |  | -5389.7833 |  | -5407.8659 |  | -5374.7177 |  |

AOR (Adjusted Odds Ratio); LL: Log-likelihood; *p-value<0.05; **p<0.001

Model 0: Empty model with no independent variables

Model 1: All environmental factors were included in the model

Model 2: Environmental factors (from model 1 with p<0.25) + Child-related factors (from model 0 with p<0.25)

Model 3: Environmental factors (from model 2 with p<0.25) + Child-related factors (from model 2 with p<0.25) + Maternal factors (from model 0 with p<0.25)

Model 4: Environmental factors (from model 3 with p<0.25) + Child-related factors (from model 3 with p<0.25) + Maternal factors (from model 3 with p<0.25)+ Household factors (from model 0 with p<0.25)

Model 5: Environmental factors (from model 4 with p<0.25) +Child related factors (from model 4 with p<0.25) + Maternal factors (from model 4 with p<0.25)+ Household factors (from model 4 with p<0.25) + Community level factors (from model 0 with p<0.25)
