# Supplementary material for: Household environment associated with anaemia among children aged 6–59 months in Ethiopia: a multilevel analysis of Ethiopia demographic and health survey (2005–2016)
Source: BMC Public Health. 2024 Jan 29;24:315. doi: 10.1186/s12889-024-17780-y (PMC10823679; doi:10.1186/s12889-024-17780-y)
Supplement: Supplementary file 4 — Additional file 4: Supplementary File 4. Adjusted association between anaemia and environmental factors and other study covariates among children 6-59 months in Ethiopia, EDHS 2016 (n=8,399). [file 12889_2024_17780_MOESM4_ESM.docx]

**Supplementary File 4: Adjusted association between anaemia and environmental factors and other study covariates among children 6-59 months in Ethiopia, EDHS 2016 (n=8,399)**

| **Variables** | **Model 0** | **Model 1** | | **Model 2** | | **Model 3** | | **Model 4** | | **Model 5** | |
| --- | --- | --- | --- | --- | --- | --- | --- | --- | --- | --- | --- |
|  | **Null model** | **AOR(95%CI)** | **p-value** | **AOR (95%CI)** | **p-value** | **AOR (95%CI)** | **p-value** | **AOR (95%CI)** | **p-value** | **AOR (95%)** | **p-value** |
| ***Environmental factors*** |  |  |  |  |  |  |  |  |  |  |  |
| **Sanitation facility** |  |  |  |  |  |  |  |  |  |  |  |
| Improved |  | Ref. |  | Ref. |  | Ref. |  | Ref. |  | Ref. |  |
| Unimproved |  | 0.88 (0.73-1.07) | 0.210 | 0.89 (0.69-1.14) | 0.374 | 0.84 (0.65-1.08) | 0.174 | 0.83 (0.64-1.06) | 0.144 | 0.85 (0.65-1.11) | 0.237 |
| Open defecation |  | 1.29 (1.06-1.57)* | 0.011 | 1.49 (1.13-1.90)* | 0.004 | 1.32 (1.01-1.73)* | 0.045 | 1.14 (0.85-1.52) | 0.370 | 1.16 (0.87-1.56) | 0.311 |
| **Source of drinking water** |  |  |  |  |  |  |  |  |  |  |  |
| Improved |  | Ref. |  |  |  |  |  |  |  |  |  |
| Unimproved |  | 1.02 (0.89-1.17) | 0.769 |  |  |  |  |  |  |  |  |
| **Time to get a water source** |  |  |  |  |  |  |  |  |  |  |  |
| On-premise |  | Ref. |  |  |  |  |  |  |  |  |  |
| ≤ 30 min |  | 1.02 (0.83-1.27) | 0.828 |  |  |  |  |  |  |  |  |
| 31-60 min |  | 1.10 (0.87-1.39) | 0.409 |  |  |  |  |  |  |  |  |
| >60 min |  | 1.01 (0.78-1.29) | 0.953 |  |  |  |  |  |  |  |  |
| **Housing status** |  |  |  |  |  |  |  |  |  |  |  |
| Built from finished materials |  | Ref. |  |  |  |  |  |  |  |  |  |
| Built from natural or unfinished materials |  | 0.96 (0.74-1.25) | 0.761 |  |  |  |  |  |  |  |  |
| **Type of cooking fuel** |  |  |  |  |  |  |  |  |  |  |  |
| Clean fuels |  | Ref. |  | Ref. |  | Ref. |  | Ref. |  | Ref. |  |
| Solid fuels |  | 1.62 (1.21-2.18) | 0.001 | 1.44 (0.99-2.11) | 0.055 | 1.34 (0.91-1.98) | 0.130 | 1.33 (0.91-1.95) | 0.139 | 1.61 (1.09-2.38)* | 0.017 |
| ***Child factors*** |  |  |  |  |  |  |  |  |  |  |  |
| **Sex** |  |  |  |  |  |  |  |  |  |  |  |
| Male |  |  |  | Ref. |  |  |  |  |  |  |  |
| Female |  |  |  | 0.96 (0.82-1.11) | 0.570 |  |  |  |  |  |  |
| **Age (months)** |  |  |  |  |  |  |  |  |  |  |  |
| 6-11 |  |  |  | 5.59 (3.05-10.2)** | p<0.001 | 5.49 (3.04-9.93)** | p<0.001 | 5.60 (3.10-10.1)** | p<0.001 | 5.52 (3.06-9.92)** | p<0.001 |
| 12-23 |  |  |  | 4.65 (2.59-8.33)** | p<0.001 | 4.58 (2.59-8.11)** | p<0.001 | 4.67 (2.65-8.25)** | p<0.001 | 4.51 (2.56-7.93)** | p<0.001 |
| 24-35 |  |  |  | 2.46 (1.38-4.39)* | 0.002 | 2.49 (1.42-4.40)** | 0.002 | 2.54 (1.44-4.46)** | 0.001 | 2.44 (1.39-4.28)** | p<0.001 |
| 36-59 |  |  |  | Ref. |  | Ref. |  | Ref. |  | Ref. |  |
| **Birth interval** |  |  |  |  |  |  |  |  |  |  |  |
| 7- 33 months |  |  |  | Ref. |  | Ref. |  | Ref. |  | Ref. |  |
| ≥ 33 months |  |  |  | 0.87 (0.73-1.03) | 0.105 | 0.79 (0.56-1.12) | 0.197 | 0.87 (0.73-1.03) | 0.114 | 0.89 (0.75-1.06) | 0.197 |
| **Size of the child at birth** |  |  |  |  |  |  |  |  |  |  |  |
| Larger |  |  |  | Ref. |  | Ref. |  | Ref. |  | Ref. |  |
| Average |  |  |  | 1.13 (0.94-1.35) | 0.183 | 1.12 (0.94-1.34) | 0.193 | 1.12 (0.94-1.34) | 0.206 | 1.14 (0.96-1.37) | 0.131 |
| Small |  |  |  | 1.34 (1.08-1.65)* | 0.006 | 1.32 (1.07-1.62)* | 0.008 | 1.31 (1.07-1.61)* | 0.009 | 1.28 (1.05-1.58)* | 0.015 |
| **Currently breastfeeding** |  |  |  |  |  |  |  |  |  |  |  |
| Yes |  |  |  | Ref. |  | Ref. |  | Ref. |  | Ref. |  |
| No |  |  |  | 1.15 (0.95-1.38) | 0.142 | 1.15 (0.96-1.39) | 0.123 | 1.16 (0.96-1.39) | 0.119 | 1.15 (0.95-1.38) | 0.144 |
| **Full vaccination** |  |  |  |  |  |  |  |  |  |  |  |
| Yes |  |  |  | Ref. |  | Ref. |  | Ref. |  | Ref. |  |
| No |  |  |  | 1.43 (1.20-1.69)** | p<0.001 | 1.36 (1.15-1.61)** | p<0.001 | 1.34 (1.13-1.58)* | 0.001 | 1.28 (1.08-1.52)* | 0.004 |
| **Received deworming medication in the last 6 months** |  |  |  |  |  |  |  |  |  |  |  |
| Yes |  |  |  | Ref. |  |  |  |  |  |  |  |
| No |  |  |  | 0.94 (0.74-1.21) | 0.658 |  |  |  |  |  |  |
| **Iron supplementation** |  |  |  |  |  |  |  |  |  |  |  |
| Yes |  |  |  | Ref. |  |  |  |  |  |  |  |
| No |  |  |  | 1.15 (0.87-1.55) | 0.320 |  |  |  |  |  |  |
| **Vitamin A last 6 months** |  |  |  |  |  |  |  |  |  |  |  |
| Yes |  |  |  | Ref. |  |  |  |  |  |  |  |
| No |  |  |  | 0.97 (0.82-1.14) | 0.705 |  |  |  |  |  |  |
| **Diarrhoea** |  |  |  |  |  |  |  |  |  |  |  |
| Yes |  |  |  | 0.95 (0.77-1.17) | 0.654 |  |  |  |  |  |  |
| No |  |  |  | Ref. |  |  |  |  |  |  |  |
| ***Parental factors*** |  |  |  |  |  |  |  |  |  |  |  |
| **Mother's age** |  |  |  |  |  |  |  |  |  |  |  |
| 15-18 |  |  |  |  |  | 0.70 (0.26-1.84) | 0.475 |  |  |  |  |
| 18-24 |  |  |  |  |  | 0.85 (0.56-1.29) | 0.454 |  |  |  |  |
| 25-34 |  |  |  |  |  | 0.94 (0.65-1.36) | 0.746 |  |  |  |  |
| 35-49 |  |  |  |  |  | Ref. |  |  |  |  |  |
| **Mother's education** |  |  |  |  |  |  |  |  |  |  |  |
| No education |  |  |  |  |  | 1.05 (0.87-1.25) | 0.619 |  |  |  |  |
| Primary and above |  |  |  |  |  | Ref. |  |  |  |  |  |
| **Mother's currently working.** |  |  |  |  |  |  |  |  |  |  |  |
| Yes |  |  |  |  |  | Ref. |  | Ref. |  | Ref. |  |
| No |  |  |  |  |  | 1.19 (1.01-1.41)* | 0.049 | 1.17 (0.98-1.39) | 0.071 | 1.16 (0.98-1.38) | 0.077 |
| **Maternal BMI (kg/m^2^)** |  |  |  |  |  |  |  |  |  |  |  |
| <18.5 |  |  |  |  |  | Ref. |  | Ref. |  | Ref. |  |
| 18.5 to 24.9 |  |  |  |  |  | 0.85 (0.71-1.03) | 0.097 | 0.86 (0.72-1.03) | 0.113 | 0.87 (0.72-1.04) | 0.134 |
| 25 + |  |  |  |  |  | 0.87 (0.63-1.20) | 0.404 | 0.89 (0.64-1.23) | 0.481 | 0.87 (0.63-1.21) | 0.419 |
| **Listening to radio** |  |  |  |  |  |  |  |  |  |  |  |
| Yes |  |  |  |  |  | Ref. |  | Ref. |  |  |  |
| Not at all |  |  |  |  |  | 1.13 (0.93-1.38) | 0.204 | 1.10 (0.91-1.33) | 0.321 |  |  |
| **Watching television** |  |  |  |  |  |  |  |  |  |  |  |
| Yes |  |  |  |  |  | Ref. |  |  |  |  |  |
| Not at all |  |  |  |  |  | 1.07 (0.84-1.36) | 0.569 |  |  |  |  |
| ***Household factors*** |  |  |  |  |  |  |  |  |  |  |  |
| **Wealth index** |  |  |  |  |  |  |  |  |  |  |  |
| Poor |  |  |  |  |  |  |  | 1.39 (1.10-1.75)* | 0.005 | 1.46 (1.16-1.86)* | 0.002 |
| Middle |  |  |  |  |  |  |  | 1.02 (0.79-1.30) | 0.889 | 1.05 (0.81-1.36) | 0.693 |
| Rich |  |  |  |  |  |  |  | Ref. |  | Ref. |  |
| ***Community-level characteristics*** |  |  |  |  |  |  |  |  |  |  |  |
| **Residence** |  |  |  |  |  |  |  |  |  |  |  |
| Urban |  |  |  |  |  |  |  |  |  | Ref. |  |
| Rural |  |  |  |  |  |  |  |  |  | 1.17 (0.85-1.59) | 0.333 |
| **Region** |  |  |  |  |  |  |  |  |  |  |  |
| Agrarian |  |  |  |  |  |  |  |  |  | Ref. |  |
| Pastoralist |  |  |  |  |  |  |  |  |  | 1.78 (1.39-2.28)** | p<0.001 |
| City administration |  |  |  |  |  |  |  |  |  | 1.95 (1.42-2.69)** | p<0.001 |
| **Random effect** |  |  |  |  |  |  |  |  |  |  |  |
| ICC (%) | 18.57 | 16.5 |  | 17.1 |  | 16.2 |  | 15.7 |  | 13.9 |  |
| Log-likelihood | -4939.1393 | -4873.3249 |  | -2347.5773 |  | -2385.7452 |  | -2381.9937 |  | -2366.2687 |  |

AOR (Adjusted Odds Ratio); LL: Log-likelihood; *p-value<0.05; **p<0.001

Model 0: Empty model with no independent variables

Model 1: All environmental factors were included in the model

Model 2: Environmental factors (from model 1 with p<0.25) + Child-related factors (from model 0 with p<0.25)

Model 3: Environmental factors (from model 2 with p<0.25) + Child-related factors (from model 2 with p<0.25) + Maternal factors (from model 0 with p<0.25)

Model 4: Environmental factors (from model 3 with p<0.25) + Child-related factors (from model 3 with p<0.25) + Maternal factors (from model 3 with p<0.25)+ Household factors (from model 0 with p<0.25)

Model 5: Environmental factors (from model 4 with p<0.25) +Child related factors (from model 4 with p<0.25) + Maternal factors (from model 4 with p<0.25)+ Household factors (from model 4 with p<0.25) + Community level factors (from model 0 with p<0.25)
